# NILs of Cold Tolerant *Japonica* Cultivar Exhibited New QTLs for Mineral Elements in Rice

**DOI:** 10.3389/fgene.2021.789645

**Published:** 2021-11-18

**Authors:** Muhammad Kazim Ali, Zheng-Hai Sun, Xiao-Meng Yang, Xiao-Ying Pu, Cheng-Li Duan, Xia Li, Lu-Xiang Wang, Jia-Zhen Yang, Ya-Wen Zeng

**Affiliations:** ^1^ Biotechnology and Germplasm Resource Institute, Yunnan Academy of Agricultural Sciences, Kunming, China; ^2^ Karachi Institute of Biotechnology and Genetic Engineering (KIBGE), University of Karachi, Karachi, Pakistan; ^3^ School of Horticulture and Gardening, Southwest Forestry University, Kunming, China; ^4^ College of Agronomy and Biotechnology, Yunnan Agricultural University, Kunming, China; ^5^ Institute of Quality Standards and Testing Technology, Yunnan Academy of Agricultural Sciences, Kunming, China

**Keywords:** chilling stress, minerals, QTLs, booting stage, tolerance

## Abstract

Chilling stress at booting stage can cause floret deterioration and sterility by limiting the supply of food chain and the accumulation of essential mineral elements resulting in reduction of yield and grain quality attributes in rice. Genomic selection of chilling tolerant rice with reference to the accumulation of mineral elements will have great potential to cope with malnutrition and food security in times of climate change. Therefore, a study was conducted to explore the genomic determinants of cold tolerance and mineral elements content in near-isogenic lines (NILs) of *japonica* rice subjected to chilling stress at flowering stage. Detailed morphological analysis followed by quantitative analysis of 17 mineral elements revealed that the content of phosphorus (P, 3,253 mg/kg) and potassium (K, 2,485 mg/kg) were highest while strontium (Sr, 0.26 mg/kg) and boron (B, 0.34 mg/kg) were lowest among the mineral elements. The correlation analysis revealed extremely positive correlation of phosphorus (P) and copper (Cu) with most of the cold tolerance traits. Among all the effective ear and the second leaf length correlation was significant with half of the mineral elements. As a result of comparative analysis, some QTLs (*qBRCC-1*, *qBRCIC-2, qBRZC-6*, *qBRCHC-6*, *qBRMC-6*, *qBRCIC-6a, qBRCIC-6b, qBRCHC-6,* and *qBRMC-6*) identified for calcium (Ca), zinc (Zn), chromium (Cr) and magnesium (Mg) on chromosome number 1, 2, and 6 while, a novel QTL (*qBCPC-1*) was identified on chromosome number 1 for P element only. These findings provided bases for the identification of candidate genes involved in mineral accumulation and cold tolerance in rice at booting stage.

## Introduction

*Oryza sativa* (an Asian cultivated rice), one of the most valuable food crops universally, is more vulnerable to freezing stress than other cereal crops such as barley (*Hordeum vulgare*) and wheat (*Triticum aestivum*), may be due to originated from subtropical or tropical zones (Sasaki and Burr, 2000; Zhang et al., 2014; Li et al., 2021). The *O. sativa* comprises of two subspecies *indica* and *japonica*, cultivated from two wild (*O. nivara* and *O. rufipogon*) rice (Sang and Ge, 2013, 2007), and are contradictory in many physiological and morphological attributes (Lv et al., 2016). Among all abiotic factors temperature is a critical environmental component influencing plant growth and development therefore *japonica* cultivars are mostly freezing tolerant, as compared to the *indica* subspecies, letting *japonica* to lead in the moderate zones. Under the pace of climate change, severe freezing climate incidents are becoming more common and low temperature reduces rice production and distribution worldwide (Jacobs and Pearson, 1994; Pan et al., 2015). Therefore, it is estimated that the losses in the production of rice in China alone are accounted for 3–5 million tons per annum (Liu and Deng, 2009) while ≥1.5 million hectares of rice producing fields are threatened due to cold stress damages. Chilling damage in rice happens at all phenological stages, together with the vegetative (germination and seedling) and reproductive (booting and flowering) phases. Chilling stress at booting stage may cause floret deterioration and sterility by limiting the food supply chain and the accumulation of essential mineral elements consequently reduction in rice yield and grain quality attributes is significant (Cruz et al., 2013). Although growth stage specific various quantitative trait loci (QTLs) have been linked with cold tolerance in rice (Zhou et al., 2010; Shinada et al., 2014; Endo et al., 2016; Li et al., 2018), only some genes have been functionally depicted, including CTB4a, Ctb1, qPSR10, qLTG3-1, HAN1, bZIP73, and COLD1 (Fujino et al., 2008; Saito et al., 2010; Ma et al., 2015; Zhang et al., 2017; Liu et al., 2018, 2019; Xiao et al., 2018; Mao et al., 2019). Because cold stress tolerance is a complicated characteristic influenced by many genes and proteins. Among all, only CTB4a and Ctb1 grant chilling tolerance at the booting stage (Saito et al., 2010; Zhang et al., 2017). Taking into account the significance of chilling tolerance in rice production, investigating more alleles/genes that can be employed to produce new cold-tolerant rice cultivars at the booting stage is highly imperative.

Essential mineral elements are more important to cope with malnutrition or hidden hanger particularly in developing countries where access to nutritious food is limited. Because food containing essential mineral elements have indirect and direct influence on the physiological and cellular metabolism of humans and plants. Therefore, the Chinese Nutrition Society recommends daily intake of some mineral elements in Chinese adults because the intake of these elements is not enough and recommended to improve the food chain to meet the requirements (Wang et al., 2017). Moreover, the poor intake of many important minerals or lack of nutritious food may cause disturbance in the function of many organs of human body which can lead to severe diseases (Sautter et al., 2006; Sun et al., 2011). Among all the nutrients, the deficiency of iron (Fe) and zinc (Zn) are the most important elements, and it affected ≥2 billion people all over the world (Kennedy et al., 2003; Hambidge and Krebs., 2007). Therefore, developmental delay and stunted growth are more common in Zn deficient patients while Fe deficient diets leads to develop anemia (Umeta et al., 2000). For example, the appropriate level of selenium level (0.3–0.5 μg·g^−1^) in rice or in its products are considered a successful way of supplying selenium for prevention of cancers (Finley et al., 2001; Sautter et al., 2006), because various epidemiological research manifested intake of selenium inversely correlates with mortality rate of cancer. Intake of approximately 400 g of se-enriched rice products per day can provide 100–200 μg of Se. Similarly, Ca is also the most essential nutrients lacking in many peoples including the Chinese people. The national per capita intake of calcium is 405 mg per day, accounting only 49.2% of Recommended Dietary Allowance (RDA) requirements (800 mg/day) therefore about 1.2 billion people are calcium deficient (Ma et al., 2005). According to World Health Organization (WHO), 50% women are iron deficient anemia in Africa and Asia. Economic loss of iron deficiency anemia (IDA) disease in China is equivalent to 3.6% of gross national product (GDP) and it is expected that the economic loss led by iron deficiency anemia in adults will reach 70 billion yuan in the next 10 years. According to another report, the total loss caused by anemia will reach 2,178.7 billion yuan (Guo et al., 2016). Likewise, Zinc is also one of the 16 essential trace elements important to human health and life. According to an estimate Zn deficiency affects the health of about two billion people around the world. Because Zn plays a catalytic or constructive role for a variety of metalloenzymes, transcription factors and proteins essential for human health (Prasad 2003; Noulas et al., 2018).

Although rice is most important staple food, but it is not a good source of minerals therefore can’t fulfill the requirements of essential microelements particularly in rural areas where people can’t afford healthy foods and they only rely on rice for their energy intake. However, there are many possible ways to developed micronutrient rich rice varieties including biofortification method to cope with malnutrition or hidden hanger (Zimmermann and Hurrell, 2002; Bouis and Welch, 2010; Bashir et al., 2013). Numerous reports on genotypic dissimilar rice accessions for accumulation of mineral elements in rice revealed indicating that the variation in the uptake and accumulation of minerals elements are species specific. Therefore, the increment of essential micronutrients in rice grain though modern breeding techniques is a vital task and the best way to cope with malnutrition (Chen et al., 2002). Currently significant attempt has been made to the enhancement of the nutritional caliber of rice grain through genetic engineering and other breeding techniques (Bao, 2014). Using mapping population of doubled haploid, many QTLs were fine mapped for Fe, Mn, P, Cu, and Zn contents (Stangoulis et al., 2007). Some other reports showed 41 QTLs for 17 mineral elements content (Norton et al., 2010). Similarly, the concentration of Ca, Fe, Mn, Cu and Zn were supposed to regulated by ten QTLs and twenty-eight interactions of digenic QTLs (Lu et al., 2008). Likewise, another report on analysis of introgression lines derived from the cross between the *Oryza ruf ipogon* (wild rice) and Teqing an *indica* elite variety manifested 31 putative QTLs for K, Mg, P, Zn, Ca, Mn, Fe, and Cu contents and among them many QTLs for these attributes were contributed by the wild rice types (Garcia-Oliveira et al., 2009). In addition, QTLs for various minerals were discovered the same position; these congregate QTLs also contribute valuable knowledge for concurrent upgrade of content of various minerals in rice kernel through molecular breeding (Ishikawa et al., 2010). For example, Zhang and his coworkers mapped about 134 trait loci (QTLs) associated with 16 minerals using two mapping populations of rice which were distributed into 39 genomic parts (Zhang et al., 2014). In another report, 14 QTLs were identified for Zn and Fe content of rice seed. While the genes (*OsNAS1, OsARD2, OsNAS2, OsIRT1,* and *OsMTP1,* and *OsYSL1*) were reported as high priority candidate genes for Zn and Fe accumulation (Anuradha et al., 2012). Elucidating the molecular markers and its expression and regulation systems for production and accumulation of essential mineral elements is obligatory for improvement of mineral elements in rice through biofortification techniques (Masuda et al., 2013). But despite the several reports, the total QTLs responsible for accumulation and distribution of mineral elements in rice grains are still need some insight particularly when plant itself are under stress condition.

Based on the importance of nutritious rice, this paper analyzes the correlation between the content of 17 mineral elements and the cold-tolerance traits of rice and aims to clarify the relationship between cold tolerance and mineral element content at the booting stage and provide a biochemical basis for the study of cold tolerance mechanism. To understand the variation and mutual relationship of mineral elements in near-isogenic lines and to provide theoretical support for improving the content of beneficial elements in brown rice. To enhance the content of mineral elements in rice grain through biofortification and influence of environmental fluctuations in this process need additional work to identify and validate new QTLs. In this study, we explored some new QTLs governing the concentration of minerals and at the same time some cold tolerance attributes using populations of NILs and the objectives were 1) to explore the genotypic variation in the content of 17 mineral elements and their correlation with phenotypic markers, 2) to identify the association of mineral elements with each other, and 3) to determine new QTLs responsible for mineral content and to elucidate the QTL combine for micronutrient elements and cold tolerance. This study should furnish the understanding of production and control of mineral elements content in rice under cold stress and may boost improvement of rice cultivars in times of climate change.

## Materials and Methods

### Plant Material

This study was carried out at two locations, namely experimental farm of Yunnan Academy of Agricultural Sciences, Kunming, China, and the second in a mountainous village of Aziying located 40 km away from Kunming, China, having 1,916 and 2,150 m altitude, respectively. Some details of the experimental condition including geographical locations, temperature, planting period, and the cold stress treatment are summarized in supporting Table 1 (Supplementary Table S1). The mapping population (261 lines including two parents) of nearly isogeneic lines (NILs) were developed as described previously by Li et al. (2018). The cold tolerant NILs progenies was obtained and selected in each backcross generation after successful crossing of cold tolerant rice cultivar (Lijing2) with cold sensitive rice cultivar (Towada). All the 261 NILs were planted in end of May and harvested in first week of October for three consecutive years (Supplementary Table S1). Standard protocols were adopted for nutrient and pest management along with conventional practices for field management and soil analysis (Supplementary Table S2) in production of rice in this area. After harvesting the rice seeds were air dried followed by dehusking on a Rice Machine (Satake Co., Tokyo, Japan).

**TABLE 1:** The mean, SD, coefficient of variation and ratio between maximum and minimum content of 17 mineral elements in the parent (Towada and Lijiang2) and in the population of near-isogenic lines (NILs) of Towada brown rice subjected to cold stress at booting stage.

Mineral element	Parental parents		Near isogenic line NIL-s			
	Towada	Lijing2	Mean	Std. D	CV (%)	Min/max
P (mg/kg)	2,977.32	3,253.23	3,376.34 ± 34.52	557.68	16.52	2020.87/5,143.92
K (mg/kg)	2,234.58	2,485.05	2,341.96 ± 16.24	262.4	11.2	1,448.95/3,113.77
Ca (mg/kg)	147.07	182.18	152.24 ± 1.24	20.01	13.14	92.07/213.38
Mg (mg/kg)	1,131.05	1,162.38	1,135.91 ± 8.82	142.56	12.55	645.92/1,580.99
S (mg/kg)	1,035.58	1,207.05	1,054.14 ± 7.56	122.1	11.58	398.55/1,370.84
Fe (mg/kg)	11.61	24.01	11.28 ± 0.19	3.00	26.64	5.40/23.62
Mn (mg/kg)	26.56	26.67	31.27 ± 0.32	5.17	16.53	16.64/54.96
Cu (mg/kg)	12.74	12.13	10.72 ± 0.42	6.79	63.32	0.00/47.60
Zn (mg/kg)	33.85	42.23	43.06 ± 0.46	7.37	17.11	23.03/67.56
B (mg/kg)	11.79	0.34	2.08 ± 0.07	1.08	51.89	0.00/9.04
Mo (mg/kg)	1.37	1.47	2.03 ± 0.08	1.24	61.28	0.00/5.08
Al (mg/kg)	7.40	6.68	10.13 ± 0.33	5.40	53.27	0.00/31.87
Cr (mg/kg)	0.93	1.05	1.29 ± 0.05	0.89	68.84	0.00/5.14
Na (mg/kg)	17.11	25.63	12.97 ± 0.77	12.44	95.93	0.00/87.43
Ni (mg/kg)	1.21	1.21	1.63 ± 0.08	1.27	77.65	0.00/6.53
Sn (mg/kg)	3.51	2.82	3.63 ± 0.12	1.96	54.02	0.00/11.85
Sr (mg/kg)	0.26	0.34	0.36 ± 0.01	0.13	36.66	0.00/0.71

### Analysis of Cold Tolerance Based on Growth and Yield Attributes

Rice adaptation under cold stress, particularly at the booting stage was assessed through number of plant growth attributes specific to booting. Therefore, rice growth attributes including anther length (AL), anther width (AW), plant height (PHt), effective tillering (ET), panicle length (PaL), flag leaf length (FLL), flag leaf width (FLW), uppermost leaf length (ULL), reciprocal first leaf length (RFLL), reciprocal first leaf width (RFLW), reciprocal secondary leaf length (RSLL), reciprocal secondary leaf width (RSLW), internode length below spike (ILBS), uppermost internode length (UIL), second internode length (SIL), first and second internode length (1-2IL), spike length (SL), full grains (FG), blighted grains (BG), and number of grain per panicle (NOGP) recorded during this study. Three individual plants per line in every repetition were noted and the mean of the two repetition (including six individual plants) as corresponding morphological traits. Full grains (FG), blighted grains (BG), number of grains per-panicle (NOGP), anther length (AL), and anther width (AW) were obtained in lab. Number of grains per panicle and rate of seed setting were determine using the following given formula.

Number of grains per panicle (NOGP) = FG + BG

Rate of seed setting (RSS) = (FG/NOGP × 100%).

Three inflorescences of individual plants per line in every repetition were measured by the Universal Projection meter and the mean of two repetitions (including 18 inflorescence), as the corresponding anther was evaluated. The correlations between RSS with 19 other morphological traits were determined by using statistical software SPSS 20.0 (SPSS Inc., Chicago, IL, United States).

### Quantitative Analysis of Mineral Elements

For further analysis of mineral elements, samples of rice seeds were smashed to powder form while the preparation of sample and quantification of mineral elements were carried out according to Jiang et al. (2007). Approximately 0.5 g of powdered rice weighed out and carbonized on an electro thermal plate at 250°C, placing it into a crucible until the sample changed into black. The samples plus crucibles were dry-ashed at 550°C for 10–12 h in a muffle furnace. A white remainder was acquired, after incineration of the sample, followed by careful shift into a volumetric flask (50 ml). Approximately 5 ml of HCl (6 M) were added in the flask to dissolve the residue and 50 ml with water were used to dilute it. The solutions (diluted) then used to determine mineral element content by inductively coupled plasma mass spectroscopy (ICP-MS) (Agilent 7500A; Agilent Technologies, CA).

### QTL Analysis

Total DNA was extracted by CTAB method using the fresh leaves of rice (Rogers and Bendich, 1989). For the design and synthesis primers, molecular markers of simple sequence repeat (SSR) were selected from the database of Gramene (http://www.gramene.org). To perform the amplification of DNA through PCR, the reaction mixtures (10 µl) was prepared with 10 µmol of forward and reverse primers, PCR buffer (10X), *Taq* DNA polymerase, dNTPs (10 mM), and 30 ng of template DNA. The condition of PCR reaction was set at: Initial denaturation at 95°C for 5 min (one cycle), followed by 35 cycles of denature at 95°C for 30 s, annealing at 55°C for 30 s and extension at 72°C for 30 s, and after these 35 cycles, reaction was set at 72°C for 10 min for final extension. To make the DNA single stranded the PCR products were subjected to 95°C for 5 min followed by electrophoresis in denaturing polyacrylamide (6%) gels followed by silver staining (Panaud et al., 1996).

According to International Rice Genome Sequencing Project (IRGSP, 2005) rice entire genome constitutes 1,526.8 cM and with mean interval cM are 2.5, and we used 647 SSR markers evenly distributed over all 12 chromosomes to assess polymorphisms between two parents. The DNA amplification followed by polyacrylamide gel electrophoresis revealed approximately 183 differential bands of SSR markers showing polymorphisms between parents and later these SSR makers used to genotype the NILs population. After performing the bulked segregation analysis (BSA), the DNA of five highest calcium containing lines were mixed and pooled as one group and the DNA content of the five lowest calcium containing lines were mixed and pooled as one group. Approximately, 125 pairs of SSR primers were used to determine the polymorphism between parents and NILs pool to identify QTLs for calcium content in brown rice seeds. There is a common banding pattern in the amplification bands of the two pools of Lijing2 and high calcium gene pools, but not in the Towada and low calcium gene pools. The selected primers are expanded to apply for the amplification of DNA of the entire population. The result of the amplification and the calcium content of brown rice was significant (One-way ANOVA). Similarly, SSR primers associated with iron and zinc were screened as described above. Analysis of QTL was carried out using QTL IciMapping 3.2 software and interval method was analyzed according to Wang et al. (2012).

### Statistical Tools for Correlation Analysis

General linear model (GLM) procedure was used to perform ANOVA test with the help of SAS program (SAS Institute, Cary, NC). To determine significant variation among NILs, a new multiple-range Duncan’s test was accomplished while PROC CORR procedure used to analysis of correlation.

## Results

### Phenotypic Traits and Correlation Analysis

Quantitative analysis of mineral elements was significantly variable between the parents and among the population. The average value, SD, coefficient of variation and minimum/maximum value of 17 mineral elements of NILs of Towada brown rice and their parents has been compiled in Table 1. Among the 17 mineral elements, phosphorus showed the highest (3,253.23 mg/kg, Lijiang2 *japonica*) amount followed by potassium (2,485.05 mg/kg, Lijiang2), while the Sr (0.26 mg/kg, Towada) and B (0.34 mg/kg, Lijiang2) had lowest in both parents and NILs. However, the content of three mineral elements (Ca, Fe, and Zn), was significantly higher in the donor (Lijiang2) parent than that of the recurrent (Towada) parent. While in the population, the coefficient of variation of Na was the largest (95.93%), followed by Ni (77.65%) and K had smallest variation (11.20%), followed by S (11.58%). Therefore, coefficient of variation of 17 elements has been found as, Na > Ni > Cr > Cu > Mo > Sn > Al > B > Fe > Zn > Sr > Mn > P > Ca > Mg > S > K. The SD showed that the distribution of P elements in the population has the largest average dispersion of the average (557.68), while the average dispersion degree of Sr element is the smallest (0.13). Therefore, the SD of the 17 elements found as: P > K > Mg > S > Ca > Na > Zn > Cu > Al > -Mn > Fe > Sn > Ni > Mo > B > Cr > Sr. Comparing the content of other elements with the content of the trace element revealed the coefficient of variation of various elements is smaller than that of trace elements while the SD is greater than that of trace elements. Comparing the differences of the 17 elements in the parents and the population, it was found that the content of the other elements except the iron element appeared in the descendant group above and below the parent, Cu, B, Mo, Al, Cr, Na. The Ni, Sn and Sr elements have undetected lines in the progeny population.

The statistical values of the kurtosis and skewness of the 17 mineral elements content in brown rice are presented in Table 2 while the normal distribution of only six minerals is presented in figures (Figure 2). Among the 17 mineral elements, ten elements (P, K, Ca, Mg, Fe, Mn, Zn, Al, Cr, and Sn) are normally distributed (KS-p >0.05), and the proportion of normal distribution of large elements (80%) is larger than that of trace elements (25%). Moreover, the maximum peak value was exhibited by B (11.86) and Na (10.24) while Zn and K showed minimum (0.01 and 0.17) peak value, respectively. Compared with kurtosis, the variation of skewness of 17 mineral elements is quite different. The five elements (K, Mg, S, Mo, and Sr) are left-biased, and the other twelve are right-biased, indicating that most of the 17 mineral elements have high content (relative and average) of the elements accounted for a large proportion. Combining the distribution of elements from parental line, in the normal distribution map, it is preliminarily concluded that the population satisfies the characteristics of the distribution of Ca, Fe, and Zn in the near isogenic lines.

**TABLE 2:** The statistical analysis (Skewness and Kurtosis) for normal distribution test of mineral elements and their results in Near-isogenic lines (NILs) of Towada brown rice.

ME	KSZ	KS-*p*	Skewness	Kurtosis
P	0.92	0.37	0.51	0.18
K	0.68	0.74	−0.13	0.17
Ca	0.57	0.90	0.15	0.21
Mg	0.42	0.99	−0.03	0.34
S	1.54	0.02	−1.74	8.83
Fe	1.45	0.05	1.07	1.88
Mn	0.66	0.77	0.48	1.51
Cu	3.59	0.00	0.53	4.82
Zn	0.68	0.75	0.31	0.01
B	2.10	0.00	2.36	11.86
Mo	1.83	0.00	−0.05	−0.97
Al	1.08	0.19	0.65	1.66
Cr	1.41	0.05	0.55	0.53
Na	3.10	0.00	2.61	10.24
Ni	2.08	0.00	0.42	−0.35
Sn	1.33	0.06	0.41	0.99
Sr	1.68	0.01	−0.65	1.34

Similarly, the correlation among 17 mineral elements quantified in brown rice of the Towada near isogenic lines are presented in supporting information (Supplementary Table S3). It is observed that among the 17 mineral elements, Fe is most closely related to the other 16 elements and showed highly positive significant with all other elements except S. The B element is the second which showed significant positive correlation with 15 elements while the P and Sr elements are significantly correlated with the 14 elements. Cu showed significant positive correlation with only seven elements, with the least of the 17 elements, followed by Zn, which is significantly correlated with the eight elements, indicating that the other elements with higher copper and zinc content are relatively less. Further analysis of the correlation between the elements found that except for the significant negative correlation between Ca and Sn elements, the others were positively correlated with each other. The supporting information (Supplementary Table S3 **)** showed that there is a correlation between most of the elements however considering the antagonistic or promoting effect of the element on absorption and accumulation, the partial correlations (Table 3) of the three elements of calcium, iron and zinc with the remaining 17 elements was calculated to eliminate the effect. Among the three elements, calcium showed extremely significant partial correlation with ten elements (P, Mg, S, Fe, Mn, Cu, Mo, Al, Cr, and Sr), in which P, Mo, Al, and Cr were negatively correlated with Ca while rest were positively correlated. Iron showed very high significant positive correlation with all elements except three (P, Ca, and Cr) elements, which showed relatively low significance level. The zinc and the six elements (Mg, S, Mo, Al, Cr, and Ni) manifested significant correlation, in which Mo and Cr were negatively correlated and the rest were positively correlated. The elements that have reached a very high significant partial correlation with iron had a very significant in simple correlation, with the difference that the coefficient of partial correlation with the three elements is reduced. The simple correlation analysis showed that the iron element and the other 16 elements have reached a high significant level and comparing the two correlations it can be concluded that iron is greatly affected by its elements. Compared with iron, the partial correlation between calcium and zinc elements and other elements is complicated. Among the ten elements that are extremely significantly related to calcium, there is no significant correlation between Cu, Mo, Al and Cr, and Ca, so the correlation coefficients of these four elements decreased. A simple comparison of the simple correlations and partial correlations of the three elements Ca, Fe, and Zn reveals that the correlation coefficient that reaches the significant or extremely significant correlation level has only a change in size, and there is no change in the relevant trend (positive and negative).

**TABLE 3:** The correlation (partial) result of calcium, iron and zinc with all 17 mineral elements content of brown rice of Towada NILs.

ME	Calcium	Iron	Zinc
P	−0.21**	0.19**	0.01
K	0.04	0.09	0.03
Ca	1.00	0.12*	0.06
Mg	0.21**	−0.04	0.25**
S	0.14*	−0.08	0.19**
Fe	0.12*	1.00	0.09
Mn	0.27**	−0.10	0.01
Cu	0.21**	0.07	0.05
Zn	0.08	0.11	1.00
B	−0.07	0.04	−0.04
Mo	−0.13*	−0.11	−0.12*
Al	−0.16**	0.15	0.13*
Cr	−0.22**	0.18**	−0.13*
Na	0.09	−0.05	−0.09
Ni	0.01	−0.03	0.12*
Sn	−0.09	0.04	0.02
Sr	0.52**	−0.02	0.08

* Means that the data is significant statistically at *p* < 0.05.

** Means it is significant at *p* < 0.01.

**FIGURE 1:**
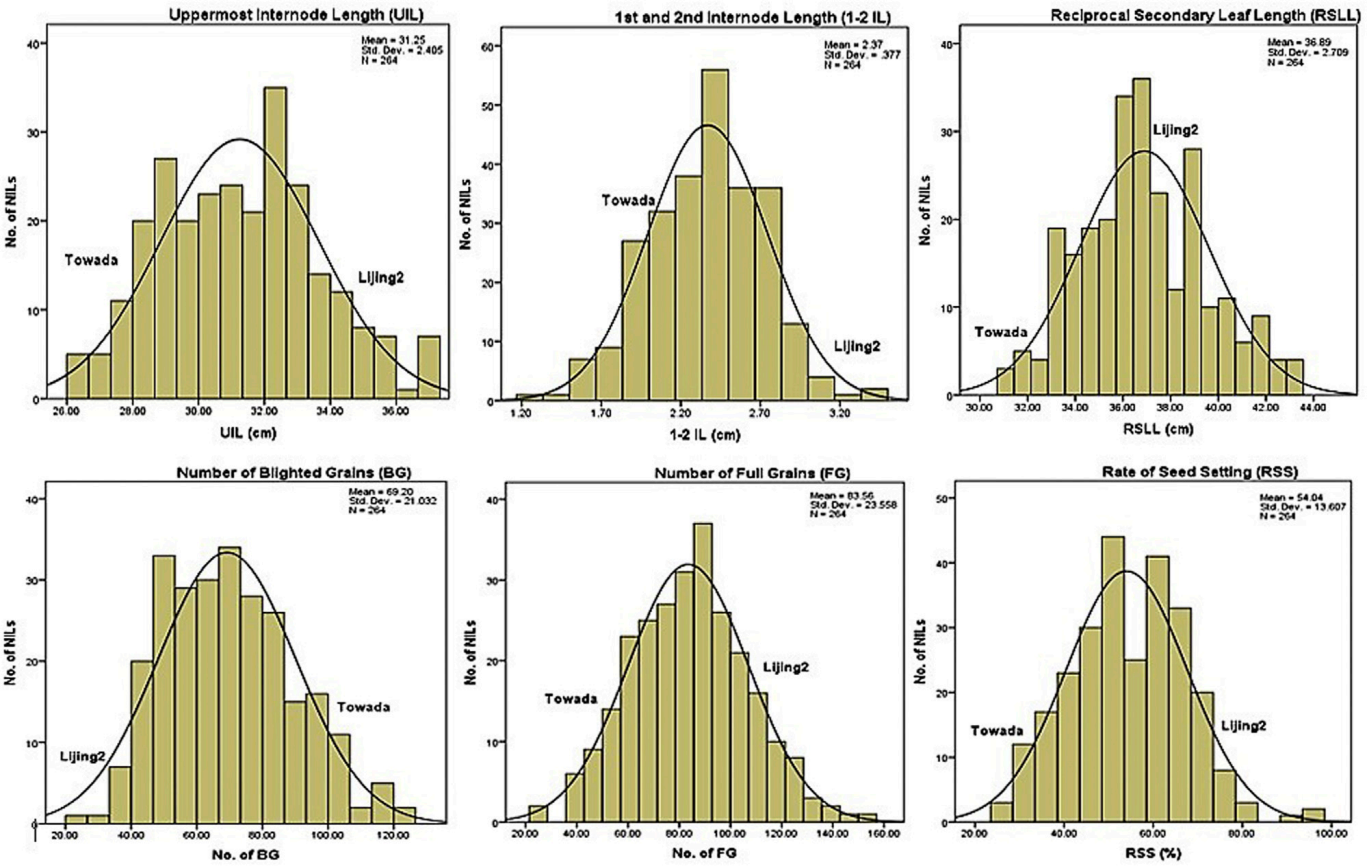
Frequency distribution of cold stress tolerance traits based on growth (UIL, 1-2 IL, RSLL) and yield (BG, FG, RSS) related attributes of NILs of Towada brown rice.

**FIGURE 2:**
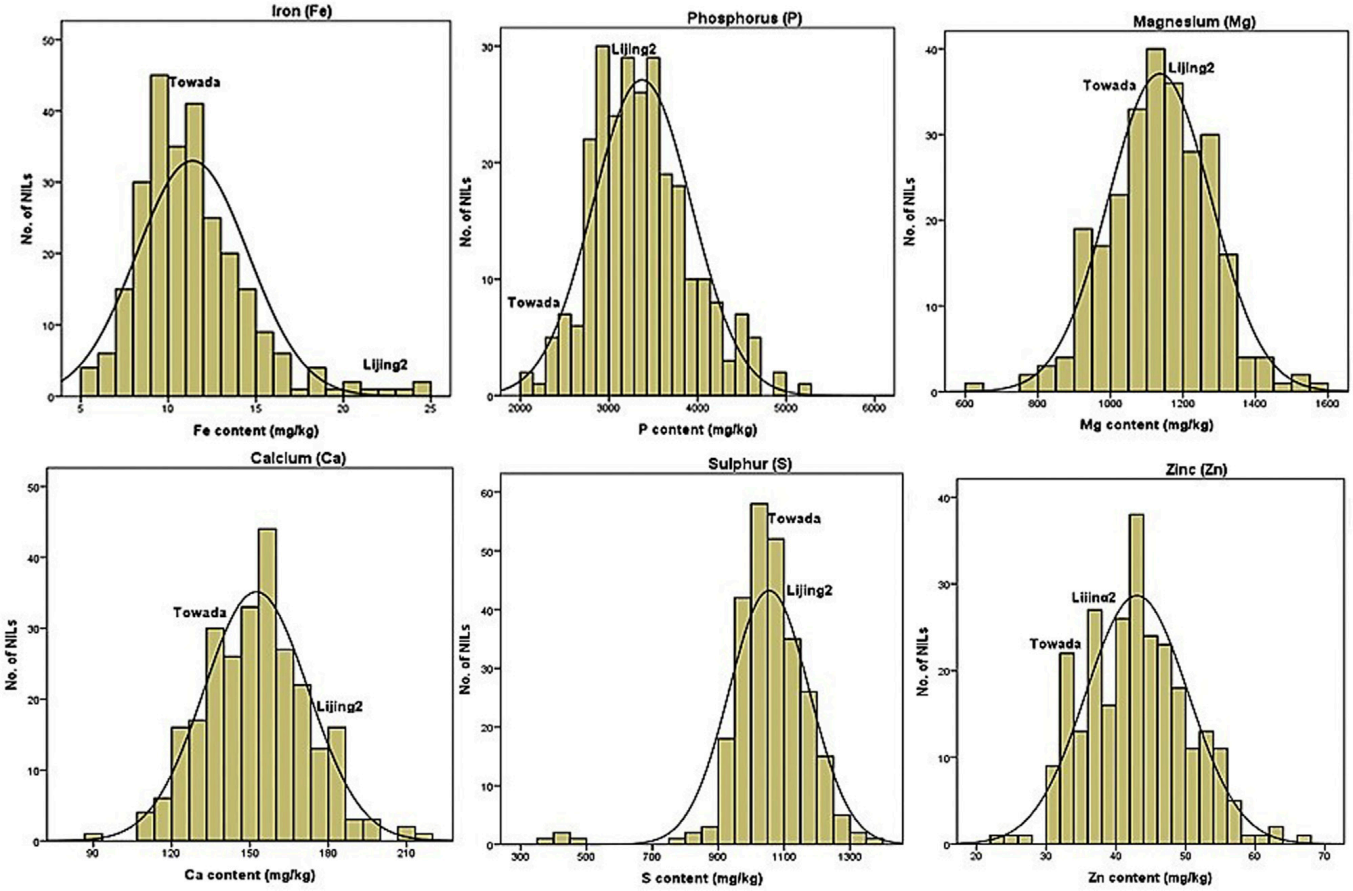
Frequency distribution of some mineral (Fe, P, Mg, Ca, S, and Zn) elements quantified in NILs of Towada brown rice subjected to chilling stress at booting stage.

Correlation analysis between mineral content and morphological traits is conducive to the selection of high (or low) elemental lines of brown rice and it supports the improvement (or reduction) of brown rice element content through cultivation measures. Correlation between the content of mineral elements in 17 brown rice varieties and other morphological traits in the Towada near-isogenic lines has been presented in the supporting file (Supplementary Table S4). Comprehensive analysis of the correlation between mineral element (17) and morphological traits (20) manifested that each element had significant correlation with only three traits (average). Among the 20 morphological traits, the effective ear and the second leaf length were most closely related to mineral elements and were significantly correlated to the seven mineral elements while the length of the ear and the length of the second internode showed correlation with six elements. However, five kinds of mineral elements had extremely significant correlation for the plant height, leaf down, while four mineral elements showed extremely significant correlation with the length and unfilled grains. While the length of the stem, the length of the flag leaf, the width of the flag leaf, the width of the inverted leaf, and the length of the 1–2 section are the least correlated with the mineral elements. No mineral element is significantly correlated to the length of the anther and the width of the inverted leaf however the number of solid grains and the seed setting rate showed significantly correlated to a mineral element. Further analysis revealed that the correlation between most mineral elements and rice anther length showed maximum correlation as compared to the width of rice grain. Similarly, anther length, one leaf length, 2 s leaf length and two internode lengths are extremely significant with P element and but with the anther width, the width of the inverted leaf and the width of the inverted two leaves, had no significant correlation. S element had significant correlation with the length of the inverted leaf, the length of the two internodes and the length of the ear. Among the 16 elements the negative correlations were more than the positive correlations and the positive/negative ratio compiled in one table (Table 4). Collectively, the correlation analysis data (Table 4 and Supplementary Table S4 of supporting file) showed that Cu is most affected by morphological traits and is significantly related to ten morphological traits, ranking first among 17 mineral elements. Secondly, P element is extremely significant and correlated significantly with eight morphological traits. Ni and Cr elements correlated significantly with six morphological traits; K element is least affected by morphological traits, and there is no form. However, the ten elements P, Mg, Fe, Mn, Cu, B, Mo, Cr, Ni, and Sn in the 17 mineral elements had significant correlation with one cold tolerance traits (Tables 4, 5). Further analysis of the above-mentioned ten elements was positively correlated with the cold-related morphological traits at the booting stage and found that except for the positive correlation with the number of glutinous grains, the others were negatively correlated, indicating that the relationship between the ten elements and the cold-tolerant traits at the booting stage was complicated. The correlation between the above elements and morphological traits was compared. The correlation coefficient between Cu and the inverted two leaves was −0.24, followed by the correlation coefficient between Fe and inverted two leaves had −0.21. In the cold-tolerant traits, the length of the second leaf was most closely related to the mineral element content and showed a very high significant correlation with the seven elements. The results of the Tables 1–5 showed that only ten of the 17 mineral elements (P, Mg, Fe, Mn, Cu, B, Mo, Cr, Ni, and Sn) are related to the cold tolerance at the booting stage. The frequency distribution of some cold tolerance (Figure 1) attributes and some minerals (Figure 2) elements are presented in the form figures. Based on this, we constructed a near-isogenic pool of brown rice calcium, iron and zinc to find its content QTL, aiming to evaluate its relationship with cold tolerance at booting stage at the molecular level and to improve the content of these beneficial mineral elements in rice.

**TABLE 4:** Relationship between the total content of 17 mineral elements and cold stress tolerance attributes of Towada brown rice NILs at booting stage.

Mineral element (ME)	Seed setting rate (SSRa)	Real grain number (FG)	Number of grains (BG)	One section length (UIL)	1-2 sections long (1-2IL)	Inverted two leaves long (RSLL)
P	−0.11	−0.06	0.16**	−0.14*	−0.02	−0.16**
K	−0.07	−0.06	0.09	−0.10	−0.03	−0.04
Ca	0.07	0.03	−0.10	0.02	0.08	−0.04
Mg	−0.11	−0.12*	0.10	−0.08	−0.01	−0.05
S	−0.08	−0.09	0.08	−0.07	0.06	−0.08
Fe	0.08	0.01	−0.07	−0.02	0.08	−0.21**
Mn	−0.08	−0.02	0.12*	−0.09	−0.03	−0.14*
Cu	0.01	−0.04	−0.03	−0.20**	−0.04	−0.24**
Zn	−0.02	−0.03	0.04	−0.11	−0.09	−0.06
B	−0.06	−0.08	−0.01	−0.03	0.01	−0.14*
Mo	−0.10	−0.07	0.12*	−0.05	−0.05	−0.06
Al	−0.05	−0.06	0.05	−0.03	−0.05	−0.10
Cr	−0.03	−0.03	0.04	−0.14*	−0.04	−0.16**
Na	0.01	−0.07	−0.08	0.06	0.04	−0.08
Ni	−0.03	−0.04	0.03	−0.13*	−0.02	−0.15*
Sn	−0.13*	−0.10	0.14*	−0.06	−0.03	−0.06
Sr	0.00	0.00	0.00	−0.02	0.00	−0.05

* Means that the data is significant statistically at *p* < 0.05.

** Means it is significant at *p* < 0.01.

**TABLE 5:** The total number of phenotypic traits linked with mineral elements content significance level of *p* 0.05 and *p* 0.01.

ME	NoPC	NoNC	ME	NoPC	NoNC
P	1	7	B	0	1
Ca	1	1	Mo	1	1
Mg	0	2	Al	0	1
S	0	3	Cr	1	5
Fe	1	2	Na	0	1
Mn	1	1	Ni	0	6
Cu	1	9	Sn	1	3
Zn	0	1	Sr	0	2

### Identification of QTLs

Screening of high calcium, iron and zinc lines were performed using SSR primers followed by synthesis of new primers for polymerase chain reaction. PCR amplification results of near-isogenic pools of Lijian2 found that primers including RM8268, RM5536, RM5644, RM5529, RM5480, RM3894, RM6364, and RM-4608, showed high band amplification in DNA samples of Lijian2 (higher mineral elements in gene pool) while there was no amplification in the DNA samples of Towada (low mineral elements in the gene pool). It is preliminarily judged that these eight primers are linked with the markers controlling to the content of calcium, iron, and zinc in Lijiang2. These eight primer pairs were used to amplify the DNA of NILs populations, and the amplification results marked as “1” common with Towada parent and “2” common with Lijing2 while both categories were marked as “3” and the missing ones marked as “0.” The amplification results then tested by one-way variance significance test with the population of calcium, iron and zinc. According to the requirement of LSD (Least-Significant Difference), the one-way variance significance test was associated with the probability value *p* at 0.05. The test results revealed only three primers (“RM5536,” “RM5529,” and “RM4608”) had significant (*p* < 0.05) variation. The amplification results of RM5536 and the one-way variance significance test of calcium, iron and zinc content showed association at *p* of 0.02, 0.05, and 0.05, respectively. The amplification results of primer RM5529 are accompanied by one-way variance significance test of calcium and iron content showed the probability values of *p* was 0.03 and 0.01, respectively. The amplification results of the primer RM4608 and the one-way variance significance test of the iron and zinc content were accompanied by probability values *p* of 0.05 and 0.05 (Table 6).

**TABLE 6:** The location of polymorphic SSR markers on chromosome and the core sequence and number of repeats with flanking regions of the polymorphic SSR markers (RM5536, RM5529, RM4608) used for primer synthesis.

S. No.	Marker name	Chrom. No.	Core Seq.	Repeats	Flanking regions
1	RM5536	1	AC	14	CAC​GTA​CCA​GCC​TTG​ATG​AAT​CC (pre) TGG​GCT​ATA​CTA​ATC​CCG​TCA​TCC (post)
2	RM5529	2	AC	13	GTA​CTA​CAT​CGG​TTG​TGT​AGT​TGG (pre) CAT​ACG​TTA​ATG​GCT​CAT​CTC​G (post)
3	RM4608	6	AT	23	ACC​CAA​TAT​GGT​GCA​ATA​GAG​ACC (pre) CAC​CTC​CAC​CAA​CTT​TGA​CAG​G (post)

Genome database search (http://www.gramene.org/) of these three SSR markers revealed that the primer “RM5536” is located on chromosome number 1 and the core sequence is 14 repeats of adenine and cytosine (AC). The specific sequence is CAC​GTA​CCA​GCC​TTG​ATG​AAT​CC (pre), TGG​GCT​ATA​CT-AAT​CCC​GTC​ATC​C (post) while the primer “RM5529” is located on chromosome 2, and the core sequence is AC with 13 repeats. The specific sequence is: GTA​CTA​CAT​CGG​TTG​TGT​AGT​TGG (pre), CAT​ACG​TTA​ATG​GCT-CAT​CTC​G (post). The primer “RM4608” was found located on chromosome 6 amplifying the 23 repeats of core sequence of AT (AT_23_), with the specific sequence: ACC​CAA​TAT​GGT-GCA​ATA​GAG​ACC (former), CAC​CTC​CAC​CAA​CTT​TGA​CAG​G (post). The content of the elements is related so these three primers can be preliminarily determined to be with calcium, iron, iron and zinc (or two of them). According to the positions of these three primers on the genetic map of rice, 10 pairs of SSR primers with similar distance were synthesized. Among them, like the primer “RM5536” the ten primers are RM5794, RM5362, RM5410, RM12171, RM12172, RM5310, RM12176, RM12177, RM12178, and RM12179. Similarly, the ten primers were synthesized as RM12406, RM12409, RM12431, RM12438, RM12440, RM12448, RM123RM12455, RM12457, and RM12466 were designed like the primer “RM5529.” While the 10 primers designed according to the primer “RM4608” were RM585, RM6536, RM1163, RM6917, RM115, RM6119, RM2434, RM7-561, RM6773, and RM2126. The newly synthesized thirty pairs of primers were subjected to PCR amplification of the near isogenic line population, and the amplification results were labeled in the same manner as above. The linkage group was constructed and confirmed using MAPMAKER 4.0 software at LOD score 3.0. 10 SSR primers synthesized and used according to RM5536 (Total 11 markers) and results showed four primers group: RM5536, RM5794, RM5362, and RM12178. Similar protocol was adopted for synthesis of new SSR markers for “RM5529” and “RM4608” also. Out of 11 primers for each the amplification results revealed five primers group including RM5529, RM12409, RM3495, RM12406, and RM12477 for former one (RM5529) and same group (five membered) including RM4608, RM19491, RM19489, RM6119, and RM19487, observed for later (RM4508) marker. A linkage group was constructed for these three groups of primers, and a linkage map was drawn using MapDraw2.1 software. Three linkage groups were analyzed and found that the primer four primers of RM5536 linkage group are biased in favor of Lijing2 and Towada and the contribution rate of the groups were 0.62 and 0.38, respectively. The five primer amplification results of the primer RM5529 linkage group, one primer (RM3495) was biased towards Lijing2, and the others were biased towards Towada. The contribution rates of Lijing2 and Towada were 0.48 and 0.52, respectively.

**FIGURE 3:**
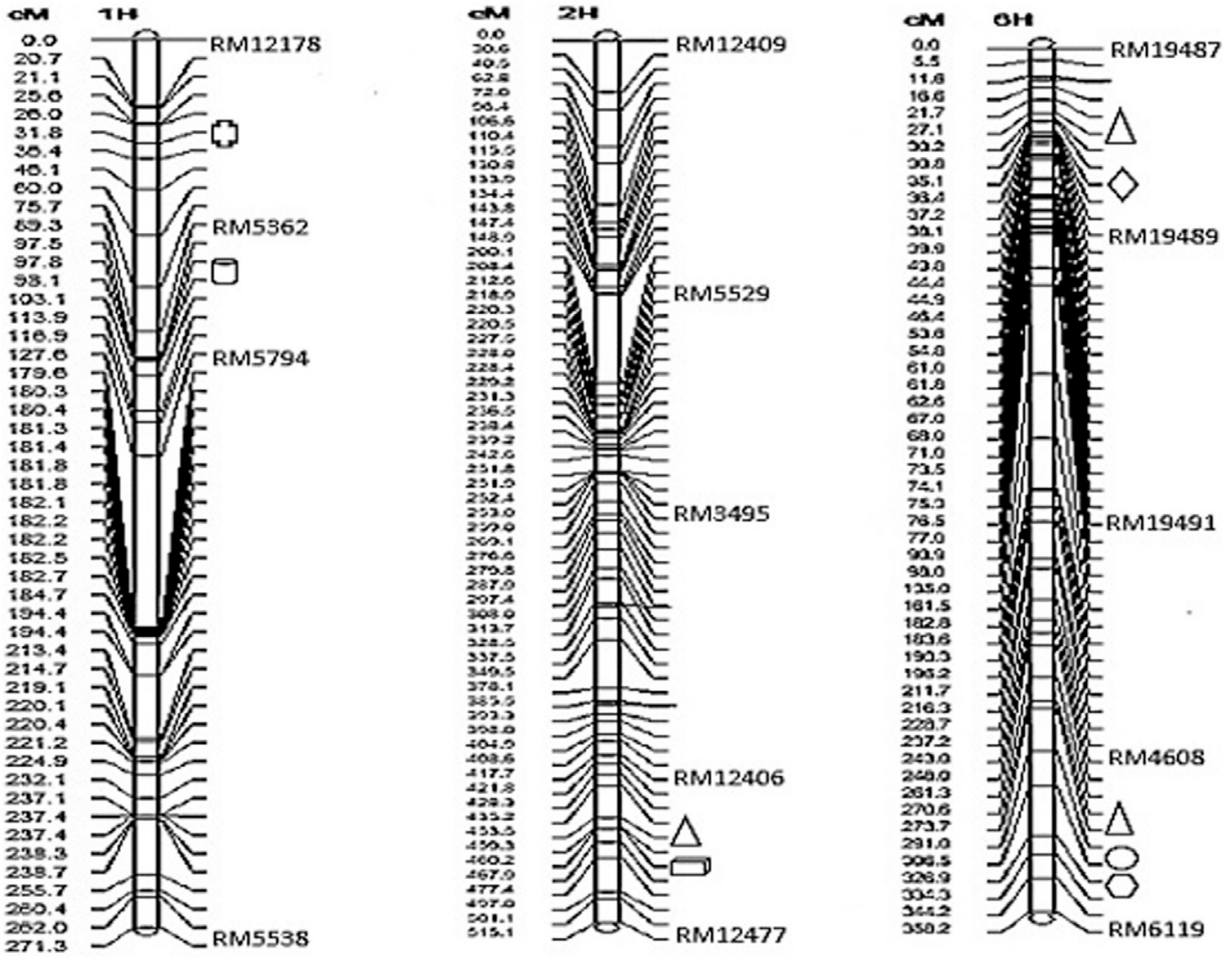
The location and congregation of QTL affecting mineral elements content in NILs of Towada brown rice. QTL for Fe (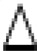), P (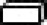), Ca (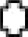), Zn (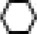), Cr (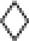), Mg (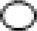), and Sr (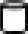) are graphically highlighted for chromosome number 1 (from left), 2 and 6.

The results of the amplification of the five primers of the primer RM4608 linkage group were all biased towards Towada, and the average contribution rate of the population of Lijing2 and Towada was 0.43 and 0.56, respectively. QTL site detection and analysis were performed on the three linkage groups established by QTLMapper1.6 software (Table 7). The table shows a QTL site for calcium located on chromosome 1 between the primers RM12178-RM5362, with −0.63 additive effect, from Lijing2 and the contribution rate was 3.95%, which could not find after querying and hence preliminarily concluded a new site and temporarily named *qBRCC-1*, according to McCouch (1997). The site controlling the zinc content was located on chromosome six in between the markers RM4608 and RM6119, and its additive effect was −1.77, from Lijing2, which explained 5.10% of the phenotypic variation. The report related to the site was found to be temporarily named *qBRZC-6*. The site controlling the Cr content of brown rice was located on chromosome 6 between RM19489-RM19491, and its additive effect was −0.26. The additive effect came from Lijing2 with a contribution rate of 8.54%. Another QTL identified responsible for the content of magnesium, and it was located between RM4608 and RM6119 on chromosome number 6, and the additive effect (−28.22) came from Lijian2, which explained 3.98% of the phenotypic variation. The two sites (Cr and Mg) temporarily named *qBRCHC-6* and *qBRMC-6*, respectively. Three QTLs found for iron content, one was located on chromosome number 2, between marker RM12406 and RM12477 and two were located on chromosome number 6, between RM1948 and RM19489 and in between RM4608 and RM6119, with −0.63, −0.79, and −0.92 additive effects Lijing2 and the contribution rates were 3.95, 5.98, and 8.24%, respectively while the cumulative contribution rate was 18.17%. After the inquiry, no relevant sites were reported, and they were initially judged to be new sites and named *qBCIC-2*, *qBCIC-6-a,* and *qBCIC-6-b*, respectively. Furthermore, another QTL was also found located on chromosome one between RM12406A-RM12477 for P content with −0.12 additive effect from Lijing2, and approximately 6.85% explain the phenotypic variation and named *qBCPC-1*. The location of identified QTLs and their intervals are also presented in graphical view of chromosome (Figure 3). While the complete information about other SSR markers between the interval markers, the type of motif, number of repeats, SSR number, forward and reverse primer, product size and start and end position are summarized in the supporting Table 5 (Supplementary Table S5).

**TABLE 7:** This tables shows the number of QTLs identified for the content of iron (Fe), phosphorous (P), calcium (Ca), strontium (Sr), chromium (Cr), zinc (Zn) and magnesium (Mg) in the brown rice of Towada NILs. It also shows the location on chromosome, intervals of markers, marker position in terms of centi-morgen (cM), the log of odd ratio (LOD) and percentage of phenotypic difference.

ME	Chr.	Interval markers	Site (cM)	LOD	Additive effect	Probability	Percentage (%) of variance explained
Fe	2	RM12406-RM12477	0.40	2.25	−0.63	0.0013	3.95
P	2	RM12406-RM12477	0.12	2.51	−0.12	0.0007	6.85
Ca	1	RM12178-RM5362	0.08	3.50	−6.23	0.0001	9.62
Sr	1	RM5362-RM5794	0.22	1.83	−0.03	0.0038	3.93
Fe	6	RM19487-RM19489	0.20	2.40	−0.79	0.0008	5.98
Fe	6	RM4608-RM6119	0.24	3.79	−0.92	0.0000	8.24
Cr	6	RM19489-RM19491	0.12	3.19	−0.26	0.0001	8.54
Zn	6	RM4608-RM6119	0.18	2.22	−1.67	0.0015	5.10
Mg	6	RM4608-RM6119	0.20	1.81	−28.22	0.0041	3.98

## Discussion

High quality seed is not only essential for human health but also ensures the maximum yield by establishing seedlings with deep roots. Therefore, the level of mineral element’s content in rice seeds is an essential element to develop high yielding yet healthy rice. Because there is a reasonable association between the quality of rice seedling and the content of mineral elements. Although some mineral elements became restrictive in natural conditions, but the 13 essential mineral elements are detrimental for growth and development of plant. Because seeds supply adequate metabolic resources to let the productive seedling establishment in the field condition. Moreover, the freshly developed seedlings moved in autotrophs condition from being heterotrophs are much depended on the reserves of mineral elements of their parent seeds (Bewley and Black, 2013). But significant variation is reported in the content of mineral element within the different genotypes of *O. sativa*. But an insight into the identification and validation of major genetic determinants for mineral elements accumulation in rice subjected to chilling stress is highly imperative. Because plants confront several abiotic stresses throughout their lifetime and extreme temperature (low/high) is a major issue. Particularly, chilling stress caused 10% reduction in rice yield per year (Wu and Garg, 2003). Because rice is more vulnerable to chilling stress than other cereal crops owing to its beginning in the hot and semitropical areas (Zhao et al., 2017). Therefore, chilling stress triggers main stress for rice growing in twenty-five states (Cruz et al., 2013) and ≥15 million ha of rice produced worldwide (Bai et al., 2016). Like other attributes, chilling stress tolerance of rice is most likely regulated by various genes depending on phenological stages (Cruz et al., 2013; Zhang et al., 2017) as well as the rice landraces. Recently He et al. (2021), reported about genetic diversity of rice landraces triggering a high degree of non-degradation adaptability to the local environment of China. According to the report, the diversity of natural and farmer choice in the course of agricultural events advances to very balanced agronomic characteristics within the population of landrace, (Pusadee et al., 2009; Song et al., 2019). Although low temperature stress tolerance related several QTLs have been mapped on all 12 chromosomes (Zeng Y. et al., 2009; Jiang et al., 2011; Kuroki et al., 2007), however only few genes (COLD1, qLTG3-1, and LTG1) conferring tolerance to low temperature at the vegetative growth stage have been isolated (Fujino et al., 2008; Ma et al., 2015). While only one gene Ctb1 (Saito et al., 2010) has been identified and cloned to confer chilling stress tolerance at the booting stage, and knowledge about the fundamental molecular mechanisms of chilling stress tolerance at the booting stage are still enigmatic. Therefore, it has been a big task to map loci linked with abiotic stress tolerance markers owing to the polygenic disposition of the loci (Shakiba et al., 2017). Kunming and Yanji (China), a high-latitude area are naturally low temperature areas, ideal for screening low temperature stress tolerance in rice (Dai et al., 2004; Blum and Tuberosa, 2018). Therefore, a study was conducted to explore the accumulation of mineral elements in brown rice at booting stage because it is a very important phenological stage. This stage enables seed production that needs epigenetic and genetic reprogramming and reassign of biochemical and metabolic resources which are highly vulnerable to chilling stress (Zhenghai et al., 2019; Jagadish et al., 2010).

Analysis of mineral elements content in the seeds of rice subjected to chilling stress revealed that phosphorus (P, 3,253 mg/kg) and potassium (K, 2,485 mg/kg) content were highest while strontium (Sr, 0.26 mg/kg) and boron (B, 0.34 mg/kg) were lowest among the 17 mineral elements. Similarly, the correlation analysis revealed extremely positive correlation of copper (Cu) and phosphorus (P) with most of the morphological traits. Similar results were reported by Bolland and Baker (1988) in which it is concluded that amount of P in the seed was positively related to the yields of annual pasture legumes. Similarly, a high content of P favored the early development of wheat seedlings as compared to plants grown at low concentration of P (Liao et al., 2008). Among the physical attributes, the effective ear and the second leaf length showed strong correlation with half of the mineral elements content. Therefore, it is preliminary concluded from the cultivation process of near isogenic lines and the distribution of calcium, iron, and zinc in the near isogenic line population, that the test population meets the requirements of brown rice high calcium, high iron, and high zinc content therefore further processed for QTL analysis. The distribution of calcium, iron and zinc in the population indicated that these elements were higher in offspring than the parental line. Moreover, all other elements showed positive correlation except Ca and Sn as reported earlier (Garcia-Oliveira et al., 2009). Approximately, 54% of the population of the brown rice showed more zinc content than Lijing2 while 37% of the brown rice had more iron and calcium content than Lijing2 indicating that the three elements are controlled by multiple genes in brown rice and there is an additive effect, which is consistent with the previous work (Pfeiffer and McClafferty, 2007; White and Brown, 2010).

Increasing the cold tolerance at the booting stage of rice can increase the yield of rice, but whether this will reduce the content of beneficial elements in rice has not been reported and only mineral elements were analyzed in the core collection of Yunnan (Zeng et al., 2010). However, correlation between morphological trait and found some elements revealed that elemental content and morphological traits were mostly negatively (Xihong et al., 2008) and the same results were obtained in this study. It is speculated that this may be related to the mechanism of elemental absorption. The agronomic traits become taller, and the anther length, ear length and leaf length are larger or wider, which means that the accumulation of elements in these organs increases, while the accumulation concentration in the grain is relatively reduced, and thus the content is relatively low. Understanding the correlation between mineral elements in brown rice has an auxiliary effect on the selection of high-mineral rice varieties of brown rice. The analysis showed high significant correlation up to 16 elements, while the least is only seven as reporter before (Zeng Y. W. et al., 2009; Huang et al., 2015; Yao et al., 2020). The antagonism or promotion of mineral elements in absorption has been confirmed in rice, such as Fe inhibits Cu and Mn absorption while it promotes Zn absorption, and Zn inhibits Cr absorption (Sasaki et al., 2016). Therefore, when performing correlation analysis between mineral elements, simple correlation and partial correlation should be analyzed simultaneously. By analyzing the simple correlation and partial correlation between the three elements of calcium, iron and zinc in brown rice and other elements manifested that the change of iron elements is relatively simple, however changes in accumulation of calcium and zinc elements are more complicated (Shao et al., 2007; Stangoulis et al., 2007).

According to the previous scientific literature, first to second node length, anther volume, number of filled grains per panicle, peduncle length, number of unfilled grains per panicle, length of the node under panicle, anther length and level of seed setting rate are phenotypic attributes strongly correlated with low temperature stress tolerance at the reproductive stage of rice (Xu et al., 2008; Shirasawa et al., 2012). Like other agronomic traits, low temperature stress tolerance is also a composite trait governed by various genes and gene products, under the influence of gene and environment. Identification and cloning of cold tolerance related QTLs have been carried out based on various morphological attributes. For example, cloning of *qLTG9* for germination of rice under cold stress, *qPSR2-1* and *qLOP2* for rice cold stress tolerance, *Hd1* controlling date of heading in rice, *TGA1* main differences in ear development between maize and teosinte, were carried out using NILs population (Cui et al., 2013; Ma et al., 2015; Jing et al., 2018). In this study, we found that Towada, are differed from its cold-sensitive recurrent parent Lijiang2 only in mean spikelet fertility after being exposed to cold stress and same were reported by Zhang et al. (2013). We performed combined analysis QTLs associated with cold tolerance and mineral elements in cold tolerant brown rice. It is observed that QTL markers interval for mineral elements (Zn, Ca, and Fe) content and QTL markers for cold tolerance interval are not on the same chromosome. QTL associated with mineral element content was reported on the chromosome number 6 while the QTL of cold tolerance was found on chromosome number 7 at booting stage in Lijian2 and Lijiang1, however there is no evidence that they have a linkage relationship. There is no correlation between calcium and zinc content in brown rice and cold tolerance at booting stage based on apparent correlation and different QTL loci. Therefore, it is preliminarily believed that there is no linkage between zinc content gene and cold tolerance gene of brown rice at booting stage. So, any improvement of mineral contents and cold tolerance in rice at booting stage may require co-localization of QTLs.

Co-localization of QTLs for various element content in seeds has formerly been stated in rice (Prasad, 2003; Stangoulis et al., 2007; Norton et al., 2010; Masuda et al., 2013; Bao, 2014). For example, co-location of QTL for Zn and Fe content was reported previously on chromosome 12 (Prasad, 2003). Previously adjacent QTLs for Zn and Fe minerals accumulations have been reported on chromosome 7 and 12 in rice, where all of Zn QTLs were co-located with the Fe QTLs except qZn7.3, suggesting possibility of selection of high Zn lines with high Fe lines using molecular (DNA) markers as selection criteria in these two regions (Masuda et al., 2013; Bao, 2014). Garcia-Oliveira et al. (2009), found 17 colocations of eight distinct minerals (K, P, Mg, Ca, Fe, Zn, Cu, and Mn), using several (85) introgression lines developed from a cross between the wild rice (*Oryza ruf* ipogon) and an elite indica cultivar Teqing. The phenomenon “pleiotropy” of the genes associated with the physiological processes and metabolism of several elements genetically found colocalization positions on chromosomes (Stangoulis et al., 2007; Norton et al., 2010). Second prospect is the occurrence of grouped genes that are strongly linked collectively and accountable for the accumulation of various elements in rice grain (Du et al., 2013). For example, the high-affinity iron regulated transporter 1 (IRT1) as a broad substrate range metal ion transporter can transport not only iron but also other divalent metals such as zinc and manganese (Du et al., 2013; Yan et al., 2015).

## Conclusion

Cold tolerance of rice and brown rice are the key to food security and human health. Brown rice is a wholegrain cereal and, as such, is known to have valuable impacts on human health. Because the brown rice is comprised of endosperm (about 90%), embryo (2–3%), and bran layers (6–7%). In addition of mineral elements, bran layer also contains bioactive molecules, such as gamma aminobutyric acid (GABA), γ-oryzanol, and ferulic acid. The nutritional value of brown rice decline under cold stress therefore it is very imperative to explore the cold tolerance and the accumulation of mineral elements in times of climate change. This study was conducted to explore the genomic determinants of cold tolerance and mineral elements content in near-isogenic lines of *japonica* rice subjected to chilling stress at booting stage. This paper not only revealed correlation between 17 mineral elements of brown rice, but also localized nine QTLs for four elements, especially a novel QTL (*qBCPC-1*) was identified on chromosome 1 for P element only. These findings provided bases for the genomic selection and identification of candidate genes involved in mineral accumulation and cold tolerance in rice which can be exploited to develop stress resilient yet healthy rice through genome editing technologies.

## Data Availability

The original contributions presented in the study are included in the article/Supplementary Material, further inquiries can be directed to the corresponding authors.

## References

[B1] Anuradha K. Agarwal S. Rao Y. V. Rao K. V. Viraktamath B. C. Sarla N. (2012). Mapping QTLs and Candidate Genes for Iron and Zinc Concentrations in Unpolished rice of Madhukar×Swarna RILs. Gene 508, 233–240. 10.1016/j.gene.2012.07.054 22964359

[B2] Bai X. Zhao H. Huang Y. Xie W. Han Z. Zhang B. (2016). Genome-wide Association Analysis Reveals Different Genetic Control in Panicle Architecture between *Indica* and *Japonica* rice. Plant Genome 9, 1–10. 10.3835/plantgenome2015.11.0115 27898816

[B3] Bao J. (2014). “Genes and QTLs for rice Grain Quality Improvement,” in Rice-germplasm, Genetics and Improvement. Editors Yan W. G. Bao J. S. (Croatia: InTech Publisher), 239–278. 10.5772/56621

[B4] Bashir K. Takahashi R. Nakanishi H. Nishizawa N. K. (2013). The Road to Micronutrient Biofortification of rice: Progress and Prospects. Front. Plant Sci. 4, 15. 10.3389/fpls.2013.00015 23404425 PMC3567483

[B5] Bewley J. D. Black M. (2013). Seeds: Physiology of Development and Germination. Springer Science & Business Media.

[B6] Blum A. Tuberosa R. (2018). Dehydration Survival of Crop Plants and its Measurement. J. Exp. Bot. 69, 975–981. 10.1093/jxb/erx445 29325054 PMC6018961

[B7] Bolland M. D. A. Baker M. J. (1988). High Phosphorus Concentrations in Seed of Wheat and Annual Medic Are Related to Higher Rates of Dry Matter Production of Seedlings and Plants. Aust. J. Exp. Agric. 28, 765–770. 10.1071/EA9880765

[B8] Bouis H. E. Welch R. M. (2010). Biofortification-a Sustainable Agricultural Strategy for Reducing Micronutrient Malnutrition in the Global South. Crop Sci. 50, S20–S32. 10.2135/cropsci2009.09.0531

[B9] Chen L. Yang F. Xu J. Hu Y. Hu Q. Zhang Y. (2002). Determination of Selenium Concentration of rice in China and Effect of Fertilization of Selenite and Selenate on Selenium Content of rice. J. Agri. Food Chem. 50, 5128–5130. 10.1021/jf0201374 12188618

[B10] Cruz R. P. D. Sperotto R. A. Cargnelutti D. Adamski J. M. de FreitasTerra T. Fett J. P. (2013). Avoiding Damage and Achieving Cold Tolerance in rice Plants. Food Energy Secur 2 (2), 96–119. 10.1002/fes3.25

[B11] Cui D. Xu C. Y. Tang C. F. Yang C. G. Yu T. Q. Xin-xiang A. (2013). Genetic Structure and Association Mapping of Cold Tolerance in Improved Japonica rice Germplasm at the Booting Stage. Euphytica 193, 369–382. 10.1007/s10681-013-0935-x

[B12] Dai L. Lin X. H. Ye C. R. Ise K. Saito K. Kato A. (2004). Identification of Quantitative Trait Loci Controlling Cold Tolerance at the Reproductive Stage in Yunnan Landrace of rice, Kunmingxiaobaigu. Breed. Sci. 54, 253–258. 10.1270/jsbbs.54.253

[B13] Du J. Zeng D. Wang B. Qian Q. Zheng S. Ling H. Q. (2013). Environmental Effects on mineral Accumulation in rice Grains and Identification of Ecological Specific QTLs. Environ. Geochem. Health 35, 161–170. 10.1007/s10653-012-9473-z 22760687

[B14] Endo T. Chiba B. Wagatsuma K. Saeki K. Ando T. Shomura A. (2016). Detection of QTLs for Cold Tolerance of rice Cultivar ‘Kuchum’ and Effect of QTL Pyramiding. Theor. Appl. Genet. 129, 631–640. 10.1007/s00122-015-2654-2 26747044

[B15] Finley J. W. Ip C. Lisk D. J. Davis D. C. Hintze K. J. Whanger P. D. (2001). Cancer-protective Properties of High-Selenium Broccoli. J. Agric. Food Chem. 49, 2679–2683. 10.1021/jf0014821 11368655

[B16] Fujino K. Sekiguchi H. Matsuda Y. Sugimoto K. Ono K. Yano M. (2008). Molecular Identification of a Major Quantitative Trait Locus, qLTG3–1, Controlling Low-Temperature Germinability in rice. Proc. Nat. Acad. Sci. 105 (34), 12623–12628. 10.1073/pnas.0805303105 18719107 PMC2527961

[B17] Garcia-Oliveira A. L. Tan L. Fu Y. Sun C. (2009). Genetic Identification of Quantitative Trait Loci for Contents of mineral Nutrients in rice Grain. J. Integr. Plant Biol. 51, 84–92. 10.1111/j.1744-7909.2008.00730.x 19166498

[B18] Guo Q. Y. Yu D. M. Yu D. Wang X. Xu X. L. Fang Y. H. (2016). Comparative Analysis of 1982, 1992, 2002, and 2010–2013 Chinese Residents Nutrition and Health Survey. J. Hyg. Res. 45, 542–547.

[B19] Hambidge K. M. Krebs N. F. (2007). Zinc Deficiency: a Special challenge. J. Nutr. 137 (4), 1101–1105. 10.1093/jn/137.4.1101 17374687

[B20] He L. Song Y. Liu X. Kang Q. Long C. (2021). Discovering Genetic Diversity of Changmaogu, a rice Landrace, for Conservation and Rural Development. Ital. J. Agron. 16 (3), 1–8. 10.4081/ija.2021.1870

[B21] Huang Y. Sun C. Min J. Chen Y. Tong C. Bao J. (2015). Association Mapping of Quantitative Trait Loci for mineral Element Contents in Whole Grain (*Oryza Sativa* L.). J. Agric. Food Chem. 63, 10885–10892. 10.1021/acs.jafc.5b04932 26641542

[B22] Ishikawa S. Abe T. Kuramata M. Yamaguchi M. Ando T. Yamamoto T. (2010). A Major Quantitative Trait Locus for Increasing Cadmium-specific Concentration in rice Grain Is Located on the Short Arm of Chromosome 7. J. Exp. Bot. 61, 923–934. 10.1093/jxb/erp360 20022924 PMC2814118

[B23] Jacobs B. Pearson C. (1994). Cold Damage and Development of rice: a Conceptual Model. Aust. J. Exp. Agric. 34, 917–919. 10.1071/ea9940917

[B24] Jagadish S. V. K. Muthurajan R. Oane R. Wheeler T. R. Heuer S. Bennett J. (2010). Physiological and Proteomic Approaches to Address Heat Tolerance during Anthesis in rice (*Oryza Sativa* L.). J. Exp. Bot. 61, 143–156. 10.1093/jxb/erp289 19858118 PMC2791117

[B25] Jiang S. L. Wu J. G. Feng Y. Yang X. E. Shi C. H. (2007). Correlation Analysis of mineral Element Contents and Quality Traits in Milled rice (*Oryza Sativa* L.). J. Agric. Food Chem. 55, 9608–9613. 10.1021/jf071785w 17937479

[B26] Jiang W. Jin Y. Lee J. Lee K. Piao R. Han L. (2011). Quantitative Trait Loci for Cold Tolerance of Rice Recombinant Inbred Lines in Low Temperature Environments. Mol. Cell 32, 579–587. 10.1007/s10059-011-0186-4 PMC3887680 22080374

[B27] Jing Y. Xiaojun N. Yaolong Y. Shan W. Qun X. Xiaoping Y. (2018). Divergent Hd1, Ghd7, and DTH7 Alleles Control Heading Date and Yield Potential of Japonica Rice in Northeast China. Front. Plant Sci. 9, 35. 29434613 10.3389/fpls.2018.00035 PMC5790996

[B28] Kennedy G. Nantel G. Shetty P. (2003). The Scourge of “Hidden Hunger”: Global Dimensions of Micronutrient Deficiencies. Food Nutr. Agric 32, 8–16.

[B29] Larcher W. (1995). Physiological Plant Ecology. Berlin: Springer-Verlag, 321–448. 10.1007/978-3-642-87851-0_6 Plants under Stress

[B30] Li J. Pan Y. Guo H. Zhou L. Yang S. Zhang Z. (2018). Fine Mapping of QTL qCTB10 2 that Confers Cold Tolerance at the Booting Stage in rice. Theor. Appl. Genet. 131, 157–166. 10.1007/s00122-017-2992-3 29032400

[B31] Li J. Zeng Y. Pan Y. Zhou L. Zhang Z. Guo H. (2021). Stepwise Selection of Natural Variations at CTB2 and CTB4a Improves Cold Adaptation during Domestication of Japonica rice. New Phytol. 231, 1056–1072. 10.1111/nph.17407 33892513

[B32] Liao M. Hocking P. J. Dong B. Delhaize E. Richardson A. E. Ryan P. R. (2008). Variation in Early Phosphorus-Uptake Efficiency Among Wheat Genotypes Grown on Two Contrasting Australian Soils. Aust. J. Agri. Res. 59 (2), 157–166. 10.1071/ar06311

[B33] Liu C. Ou S. Mao B. Tang J. Wang W. Wang H. (2018). Early Selection of bZIP73 Facilitated Adaptation of Japonica rice to Cold Climates. Nat. Commun. 9, 3302. 10.1038/s41467-018-05753-w 30120236 PMC6098049

[B34] Liu C. Schlappi M. R. Mao B. Wang W. Wang A. Chu C. (2019). The bZIP73 Transcription Factor Controls rice Cold Tolerance at the Reproductive Stage. Plant Biotech. J. 17, 1834–1849. 10.1111/pbi.13104 PMC6686130 30811812

[B35] Liu Z. Deng H. (2009). Development of Genetic and QTLs Analysis for Cold Tolerance in rice. Chin. Agri. Sci. Bull. 25, 45–50.

[B36] Lu K. Li L. Zheng X. Zhang Z. Mou T. Hu Z. (2008). Quantitative Trait Loci Controlling Cu, Ca, Zn, Mn and Fe Content in rice Grains. J. Genet. 87 (3), 305–310. 10.1007/s12041-008-0049-8 19147920

[B37] Lv Y. Guo Z. Li X. Ye H. Li X. Xiong L. (2016). New Insights into the Genetic Basis of Natural Chilling and Cold Shock Tolerance in rice by Genome-wide Association Analysis. Plant Cel Environ 39, 556–570. 10.1111/pce.12635 26381647

[B38] Ma G. Jin Y. Piao J. Kok F. Guusje B. Jacobsen E. (2005). Phytate, Calcium, Iron, and Zinc Contents and Their Molar Ratios in Foods Commonly Consumed in China. J. Agric. Food Chem. 53 (26), 10285–10290. 10.1021/jf052051r 16366728

[B39] Ma Y. Dai X. Xu Y. Luo W. Zheng X. Zeng D. Chong K. (2015). COLD1 Confers Chilling Tolerance in rice. Cell 160 (6), 1209–1221. 10.1016/j.cell.2015.01.046 25728666

[B40] Mao D. Xin Y. Tan Y. Hu X. Bai J. Liu Z. (2019). Natural Variation in the *HAN1* Gene Confers Chilling Tolerance in rice and Allowed Adaptation to a Temperate Climate. PNAS, USA 116, 3494–3501. 10.1073/pnas.1819769116 30808744 PMC6397538

[B41] Masuda H. Aung M. S. Nishizawa N. K. (2013). Iron Biofortification of rice Using Different Transgenic Approaches. Rice 6 (1), 1–12. 10.1186/1939-8433-6-40 24351075 PMC3878263

[B42] McCouch S. R. (1997). Report on QTL Nomenclature. Rice Genet. Newsl. 14, 11–13.

[B43] Norton G. J. Deacon C. M. Xiong L. Huang S. Meharg A. A. Price A. H. (2010). Genetic Mapping of the rice Ionome in Leaves and Grain: Identification of QTLs for 17 Elements Including Arsenic, Cadmium, Iron and Selenium. Plant Soil 329 (1), 139–153. 10.1007/s11104-009-0141-8

[B44] Noulas C. Tziouvalekas M. Karyotis T. (2018). Zinc in Soils, Water and Food Crops. J. Trace. Elem. Med. Biol. 49, 252–260. 10.1016/j.jtemb.2018.02.009 29472130

[B45] Pan Y. Zhang H. Zhang D. Li J. Xiong H. Yu J. (2015). Genetic Analysis of Cold Tolerance at the Germination and Booting Stages in rice by Association Mapping. PLoS ONE 10, e0120590. 10.1371/journal.pone.0120590 25790128 PMC4366098

[B46] Panaud O. Chen X. McCouch S. R. (1996). Development of Microsatellite Markers and Characterization of Simple Sequence Length Polymorphism (SSLP) in rice (*Oryza Sativa* L.). Mol. Gen. Genet. 252, 597–607. 10.1007/bf02172406 8914521

[B47] Pfeiffer W. H. McClafferty B. (2007). HarvestPlus: Breeding Crops for Better Nutrition. Crop Sci., 47, S-88. 10.2135/cropsci2007.09.0020ipbs

[B48] Prasad A. S. (2003). Zinc Deficiency Has Been Known of for 40 Years but Ignored by Global Health Organizations. B. M. J. 22, 409–410. 10.1136/bmj.326.7386.409

[B49] Pusadee T. Jamjod S. Chiang Y. Rerkasem B. Schaal B. (2009). Genetic Structure and Isolation by Distance in a Landrace of Thai rice. Proc. Natl. Acad. Sci. U. S. A. 106, 13880–13885. 10.1073/pnas.0906720106 19651617 PMC2728989

[B50] Rogers S. O. Bendich A. J. (1989). Extraction of DNA from Plant tissuesPlant Molecular Biology Manual. New York: Springer, 73–83. 10.1007/978-94-009-0951-9_6

[B51] Saito K. Hayano Y. Kuroki M. Sato Y. (2010). Map-based Cloning of the rice Cold Tolerance Gene Ctb1. Plant Sci. 179, 97–102. 10.1016/j.plantsci.2010.04.004

[B52] Sang T. Ge S. (2007). Genetics and Phylogenetics of rice Domestication. Curr. Opin. Genet. Dev. 17, 533–538. 10.1016/j.gde.2007.09.005 17988855

[B53] Sang T. Ge S. (2013). Understanding rice Domestication and Implications for Cultivar Improvement. Curr. Opin. Plant Biol. 16, 139–146. 10.1016/j.pbi.2013.03.003 23545218

[B54] Sasaki A. Yamaji N. Ma J. F. (2016). Transporters Involved in mineral Nutrient Uptake in rice. J. Exp. Bot. 67, 3645–3653. 10.1093/jxb/erw060 26931170

[B55] Sasaki T. Burr B. (2000). International rice Genome Sequencing Project: the Effort to Completely Sequence the rice Genome. Curr. Opin. Plant Biol. 3, 138–142. 10.1016/s1369-5266(99)00047-3 10712951

[B56] Sautter C. Poletti S. Zhang P. Gruissem W. (2006). Biofortification of Essential Nutritional Compounds and Trace Elements in rice and Cassava. Proc. Nutr. Soc. 65, 153–159. 10.1079/pns2006488 16672076

[B57] Shakiba E. Edwards J. D. Jodari F. Duke S. E. Baldo A. M. Korniliev P. (2017). Genetic Architecture of Cold Tolerance in rice (*Oryza Sativa*) Determined through High Resolution Genome-wide Analysis. PLoS One 12. 10.1371/journal.pone.0172133 PMC5345765 28282385

[B58] Shao H. B. Guo Q. J. Chu L. Y. Zhao X. N. Su Z. L. Hu Y. C. (2007). Understanding Molecular Mechanism of Higher Plant Plasticity under Abiotic Stress. Colloids Surf. B: Biointerfaces 54 (1), 37–45. 10.1016/j.colsurfb.2006.07.002 16914294

[B59] Shinada H. Iwata N. Sato T. Fujino K. (2014). QTL Pyramiding for Improving of Cold Tolerance at Fertilization Stage in rice. Breed. Sci. 63, 483–488. 10.1270/jsbbs.63.483 24757388 PMC3949585

[B60] Shirasawa S. Endo T. Nakagomi K. Yamaguchi M. Nishio T. (2012). Delimitation of a QTL Region Controlling Cold Tolerance at Booting Stage of a cultivar,‘Lijiangxintuanheigu’, in rice, *Oryza Sativa* L. Theor. Appl. Genet. 124, 937–946. 10.1007/s00122-011-1758-6 22113591

[B61] Song Y. J. Qiong F. Jarvis D. Bai K. Liu D. M. Feng J. C. (2019). Network Analysis of Seed Flow, a Traditional Method for Conserving Tartary Buckwheat (*Fagopyrum Tataricum*) Landraces in Liangshan, Southwest China. Sustain.-Basel. 11, 4263. 10.3390/su11164263

[B62] Stangoulis J. C. Huynh B. L. Welch R. M. Choi E. Y. Graham R. D. (2007). Quantitative Trait Loci for Phytate in rice Grain and Their Relationship with Grain Micronutrient Content. Euphytica 154 (3), 289–294. 10.1007/s10681-006-9211-7

[B63] Sun M. M. Abdula S. E. Lee H. J. Cho Y. C. Han L. Z. Koh H. J. (2011). Molecular Aspect of Good Eating Quality Formation in Japonica rice. PLoS One 6 (4), e18385. 10.1371/journal.pone.0018385 21494675 PMC3071818

[B64] Sun Z. Du J. Pu X. Kazim Ali M. Yang X. Duan C. Zeng Y. (2019). Near-isogenic Lines of Japonica rice Revealed New QTLs for Cold Tolerance at Booting Stage. Agronomy 9 (1), 40. 10.3390/agronomy9010040

[B65] Umeta M. West C. E. Haidar J. Deurenberg P. Hautvast J. G. (2000). Zinc Supplementation and Stunted Infants in Ethiopia: a Randomised Controlled Trial. The lancet 355 (9220), 2021–2026. 10.1016/s0140-6736(00)02348-5 10885352

[B66] Wang H. Wang D. Ouyang Y. Huang F. Ding G. Zhang B. (2017). Do Chinese Children Get Enough Micronutrients? Nutrients 9 (4), 397. 10.3390/nu9040397 PMC5409736 28420205

[B67] Wang J. K. Li H. H. Zhang L. Y. Meng L. (2012). QTL IciMapping Version 3.2. Beijing, China: Institute of Crop Sciences, Chinese Academy of Agricultural Sciences.

[B68] White P. J. Brown P. H. (2010). Plant Nutrition for Sustainable Development and Global Health. Ann. Bot. 105, 1073. 10.1093/aob/mcq085 20430785 PMC2887071

[B69] Wu R. Garg A. (2003). ISB News Report, March. Covering Agricultural and Environmental Biotechnology Developments. February: ISB News Report.Engineering rice Plant with Trehalose Producing Genes Improves Tolerance to Drought, Salt and Low Temperature

[B70] Xiao N. Gao Y. Qian H. Gao Q. Wu Y. Zhang D. (2018). Identification of Genes Related to Cold Tolerance and a Functional Allele that Confers Cold Tolerance. Plant Physiol. 177, 1108–1123. 10.1104/pp.18.00209 29764927 PMC6052991

[B71] Xihong S. Shenguang C. Liyong C. (2008). Construction of Genetic Linkage Map Based on a RIL Population Derived from Super Hybrid rice, XY9308. Mol. Plant Breed. 6, 861–866.

[B72] Xu L. M. Zhou L. Zeng Y. W. Wang F. M. Zhang H. L. Shen S. Q. (2008). Identification and Mapping of Quantitative Trait Loci for Cold Tolerance at the Booting Stage in a Japonica rice Near-Isogenic Line. Plant Sci. 174 (3), 340–347. 10.1016/j.plantsci.2007.12.003

[B73] Yan H. Chengxiao S. Jie M. Yaling C. Chuan T. Jinsong B. (2015). Association Mapping of Quantitative Trait Loci for Mineral Element Contents in Whole Grain Rice (*Oryza Sativa* L.). J. Agric. Food Chem. 63, 10885–10892. 10.1021/acs.jafc.5b04932 26641542

[B74] Yao B. M. Chen P. Sun G. X. (2020). Distribution of Elements and Their Correlation in Bran, Polished rice, and Whole Grain. Food Sci. Nutr. 8 (2), 982–992. 10.1002/fsn3.1379 32185023 PMC7075078

[B75] Ye C. Fukai S. Godwin I. Reinke R. Snell P. Schiller J. (2009). Cold Tolerance in rice Varieties at Different Growth Stages. Crop Pasture Sci. 60 (4), 328–338. 10.1071/cp09006

[B76] Zeng Y. W. Wang L. X. Du J. Wang S. M. Yang Y. C. Li Q. W. Du W. (2009b). Correlation of mineral Elements between Milled and Brown rice and Soils in Yunnan Studied by ICP-AES. Spectrosc. Spectral Anal. 29 (5), 1413–1417. 19650503

[B77] Zeng Y. Yang S. Cui H. Yang X. Xu L. Du J. (2009a). QTLs of Cold Tolerance-Related Traits at the Booting Stage for NIL-RILs in rice Revealed by SSR. Genes Genom 31 (2), 143–154. 10.1007/bf03191147

[B78] Zeng Y. Zhang H. Wang L. Pu X. Du J. Yang S. (2010). Genotypic Variation in Element Concentrations in Brown rice from Yunnan Landraces in China. Environ. Geochem. Health 32, 165–177. 10.1007/s10653-009-9272-3 19554457

[B79] Zhang M. Pinson S. R. Tarpley L. Huang X. Y. Lahner B. Yakubova E. Salt D. E. (2014). Mapping and Validation of Quantitative Trait Loci Associated with Concentrations of 16 Elements in Unmilled rice Grain. Theor. Appl. Genet. 127, 137–165. 10.1007/s00122-013-2207-5 24231918 PMC4544570

[B80] Zhang Q. Jiang N. Wang G. L. Hong Y. Wang Z. (2013). Advances in Understanding Cold Sensing and the Cold-Responsive Network in rice. Adv. Crop Sci. Tech. 1 (1), 104. 10.4172/2329-8863.1000104

[B81] Zhang Z. Li J. Pan Y. Li J. Shi H. Zeng Y. (2017). Natural Variation in CTB4a Enhances rice Adaptation to Cold Habitats. Nat. Commun. 8 (1), 1–13. 10.1038/ncomms14788 28332574 PMC5376651

[B82] Zhao J. Zhang S. Dong J. Yang T. Mao X. Liu Q. (2017). A Novel Functional Gene Associated with Cold Tolerance at the Seedling Stage in rice. Plant Biotechnol. J. 15, 1141–1148. 10.1111/pbi.12704 28173633 PMC5552475

[B83] Zhou L. Zeng Y. Zheng W. Tang B. Yang S. Zhang H. (2010). Fine Mapping a QTL qCTB7 for Cold Tolerance at the Booting Stage on rice Chromosome 7 Using a Near-Isogenic Line. Theor. Appl. Genet. 121, 895–905. 10.1007/s00122-010-1358-x 20512559

[B84] Zimmermann M. B. Hurrell R. F. (2002). Improving Iron, Zinc and Vitamin A Nutrition through Plant Biotechnology. Curr. Opin. Biotechnol. 13 (2), 142–145. 10.1016/s0958-1669(02)00304-x 11950566

