# Supplementary material for: NILs of Cold Tolerant Japonica Cultivar Exhibited New QTLs for Mineral Elements in Rice
Source: Front Genet. 2021 Nov 18;12:789645. doi: 10.3389/fgene.2021.789645 (PMC8637755; doi:10.3389/fgene.2021.789645)
Supplement: Supplementary file 1 [file DataSheet1.docx]

**Supplementary Tables**

**Table S1.** Some information about experimental design and field condition

|  | **Experimental locations** | |
| --- | --- | --- |
|  | **Kunming** | **Aziying** |
| Number of lines | 261×2 | 261×2 |
| Sowing date | Mid-March | Mid-March |
| Transplant date | End of May | End of May |
| Harvest date | Early October | Early October |
| Average temperature | 16-18℃ | 15-18℃ |
| GPS Coordinates  Cold treatment method | 24.8801° N, 102.8329° E  Cool-water irrigation system | 25.2030° N, 102.4643° E  Cool-water irrigation system |
| Duration of booting to maturity | July to August | July to August |
| Air temperature (booting stage) | 19.0℃ | 17℃ |
| Water temperature | 18.5±0.50 | 17.4±0.30 |

**Table S2.** Average value (mg/kg) of soil nutrients (Minerals) of the rice growing field at two locations. The elements are Sulphur (S), Selenium (Se), Molybdenum (Mo), Barium (Ba), Iren (Fe), Chromium (Cr), Sodium (Na), Aluminum (Al), Copper (Cu), Phosphorous (P), Stannum (Sn), Zinc (Zn), Boron (B), Manganese (Mn), Magnesium (Mn), Calcium (Ca) and Potassium (K).

| **Location** | **S** | **Se** | **Mo** | **Ba** | **Ni** | **Fe** | **Cr** | **Na** | **Al** |
| --- | --- | --- | --- | --- | --- | --- | --- | --- | --- |
| **Kunming** | 604.1 | 127.4 | 43.8 | 147.6 | 55.3 | 58604.5 | 69.3 | 200.4 | 36958.2 |
| **Aziying** | 608.0 | 101.9 | 39.1 | 171.8 | 50.2 | 54200.4 | 69.3 | 236.3 | 41898.6 |
|  | **Cu** | **P** | **Sn** | **Zn** | **B** | **Mn** | **Mg** | **Ca** | **K** |
| **Kunming** | 101.5 | 1320.9 | 46.6 | 143.2 | 360.7 | 527.1 | 5031.1 | 4639.9 | 6662.0 |
| **Aziying** | 96.9 | 1275.5 | 39.8 | 101.8 | 367.6 | 532.7 | 5499.3 | 4481.7 | 8537.2 |

**Table S3:** Correlation among seventeen mineral elements quantified in the brown rice of Towada NILs under cold stress.

| **ME** | | **P** | **K** | **Ca** | **Mg** | **S Fe** | | **Mn** | | **Cu** | **Zn** | | **B** | **Mo** | **Al** | **Cr** | | **Na** |  | | **Ni** | **Sn** |  |  |
| --- | --- | --- | --- | --- | --- | --- | --- | --- | --- | --- | --- | --- | --- | --- | --- | --- | --- | --- | --- | --- | --- | --- | --- | --- |
| **K** | | 0.69** | |  |  |  |  |  |  |  | |  |  | |  |  | |  |  | |  | |  | |
| **Ca** | | 0.29 | | 0.53* |  |  |  |  |  |  | |  |  | |  |  | |  |  | |  | |  | |
| **Mg** | | 0.80** | | 0.87** | 0.53 |  |  |  |  |  | |  |  | |  |  | |  |  | |  | |  | |
| **S** | | 0.54* | | 0.29 | 0.17 | 0.46* |  |  |  |  | |  |  | |  |  | |  |  | |  | |  | |
| **Fe** | | 0.39* | | 0.36* | 0.27* | 0.34* | 0.15 |  |  |  | |  |  | |  |  | |  |  | |  | |  | |
| **Mn** | | 0.57* | | 0.59* | 0.46* | 0.57* | 0.26* | 0.28* |  |  | |  |  | |  |  | |  |  | |  | |  | |
| **Cu** | | 0.02 | | 0.01 | -0.02 | -0.02 | 0.07 | 0.48* | 0.07 |  | |  |  | |  |  | |  |  | |  | |  | |
| **Zn** | | 0.55* | | 0.60** | 0.47* | 0.68** | 0.41* | 0.31* | 0.41* | 0.00 | |  |  | |  |  | |  |  | |  | |  | |
| **B** | | 0.20 | | 0.23* | 0.27* | 0.28* | 0.38* | 0.16 | 0.28** | 0.04 | | 0.24* |  | |  |  | |  |  | |  | |  | |
| **Mo** | | 0.25 | | -0.05 | -0.11 | -0.01 | 0.20* | 0.23* | 0.14 | 0.17 | | -0.08 | 0.25* | |  |  | |  |  | |  | |  | |
| **Al** | | 0.20 | | 0.05 | 0.1 | 0.04 | 0.04 | 0.38* | 0.19 | 0.20* | | 0.05 | 0.24* | | 0.72** |  | |  |  | |  | |  | |
| **Cr** | | 0.18 | | 0.00 | -0.03 | -0.02 | 0.12 | 0.50* | 0.15 | 0.69** | | -0.05 | 0.21* | | 0.76** | 0.68** | |  |  | |  | |  | |
| **Na** | | 0.01 | | 0.06 | 0.22* | 0.03 | 0.06 | 0.16* | 0.01 | 0.08 | | 0.01 | 0.30* | | 0.34* | 0.49* | | 0.35* |  | |  | |  | |
| **Ni** | | 0.19 | | -0.05 | -0.05 | -0.04 | 0.18 | 0.38* | 0.16 | 0.47* | | -0.06 | 0.26* | | 0.90** | 0.72** | | 0.92** | 0.37* | |  | |  | |
| **Sn** | | 0.23 | | -0.1 | -0.15 | -0.05 | 0.17 | 0.20* | 0.08 | 0.22* | | -0.1 | 0.11 | | 0.91** | 0.59* | | 0.68** | 0.24* | | 0.80** | |  | |
| **Sr** | | 0.18 | | 0.15 | 0.37* | 0.12 | 0.02 | 0.36* | 0.24* | 0.13* | | 0.11 | 0.35* | | 0.67** | 0.79** | | 0.65** | 0.54* | | 0.68** | | 0.53* | |

| **Morph. Trait** | **P** | **K** | **Ca** | **Mg** | **S** | **Fe** | **Mn** | **Cu** | **Zn** | **B** | **Mo** | **Al** | **Cr** | **Na** | **Ni** | **Sn** | **Sr** |
| --- | --- | --- | --- | --- | --- | --- | --- | --- | --- | --- | --- | --- | --- | --- | --- | --- | --- |
| Anther length | -.12* | -0.06 | -0.03 | -0.09 | -0.11 | 0.05 | -0.02 | 0.05 | -0.11 | -0.05 | -0.07 | -0.06 | -0.02 | 0.00 | -0.04 | -0.04 | -0.05 |
| Anther width | -0.04 | -0.05 | -0.07 | -0.04 | 0 | 0.14* | -0.05 | 0.20** | 0.01 | 0.01 | 0.04 | 0.00 | 0.12* | 0.00 | 0.10 | 0.06 | -0.01 |
| Plant height | -0.06 | 0.01 | 0.06 | 0.04 | -0.09 | -0.10 | -0.02 | -.19** | -0.07 | -0.04 | -0.10 | -0.07 | -0.14* | -0.05 | -0.13* | -0.13* | 0.02 |
| Effective spike | -.17** | -0.01 | -0.01 | -0.04 | -0.12 | -0.01 | -0.11 | -0.13* | -0.05 | -0.07 | -0.15* | -0.12 | -0.17** | 0.00 | -.22** | -0.15* | -0.15* |
| Ear length | 0.00 | 0.01 | 0.07 | 0.02 | 0.03 | 0.00 | 0.07 | 0.02 | -0.07 | 0.01 | 0.04 | 0.00 | 0.04 | 0.07 | 0.04 | 0.03 | 0.06 |
| Sword leaf length | -0.1 | 0.08 | 0.07 | 0.03 | -0.11 | 0.10 | 0.01 | -0.04 | 0.05 | 0.01 | -0.05 | -0.06 | -0.03 | 0.03 | -0.08 | -0.06 | 0.04 |
| Blade leaf width | -0.01 | -0.06 | -0.13* | -0.03 | 0.01 | -0.09 | -0.10 | -0.11 | -0.01 | -0.09 | 0.02 | -0.08 | -0.05 | -0.07 | -0.05 | 0.03 | -0.08 |
| Ear section | -0.02 | -0.01 | 0.00 | 0.04 | 0.06 | -0.09 | 0.00 | -.25** | -0.02 | -0.08 | -0.10 | -0.17** | -0.23** | 0.00 | -.22** | -0.09 | -.21** |
| Pour a long leaf | -0.14* | 0.04 | 0.02 | -0.03 | -.22** | -0.12* | -0.02 | -0.14* | -0.07 | -0.05 | -0.02 | -0.04 | -0.06 | 0.05 | -0.07 | -0.04 | 0.04 |
| Invert. leaf width | 0.01 | 0.05 | -0.08 | 0.01 | 0.01 | -0.03 | 0.02 | -0.03 | 0.03 | -0.10 | 0.05 | 0.00 | 0.02 | -0.05 | 0.01 | 0.04 | -0.03 |
| Total no. of grains | 0.11 | 0.03 | -0.08 | -0.04 | -0.02 | -0.07 | 0.11 | -0.09 | 0.00 | -0.10 | 0.05 | -0.01 | 0.00 | -0.18** | -0.01 | 0.03 | -0.01 |
| Invert. 2 leaf long | -.16** | -0.04 | -0.04 | -0.05 | -0.08 | -.21** | -0.14* | -.24** | -0.06 | -0.14* | -0.06 | -0.10 | -0.16** | -0.08 | -0.15* | -0.06 | -0.05 |
| Invert. leaf width | -0.04 | -0.01 | -0.04 | -0.05 | -0.03 | -0.01 | -0.04 | -0.13* | -0.04 | -0.01 | 0.10 | 0.06 | 0.02 | 0.03 | 0.06 | 0.10 | 0.05 |
| 2 internode length | -.12* | 0.03 | 0.13* | 0.03 | -0.13* | -0.09 | -0.06 | -0.13* | -0.05 | 0.03 | -0.09 | -0.04 | -0.11 | -0.01 | -0.15* | -0.1 | 0.04 |
| Spike length | -.17** | -0.11 | -0.1 | -0.15* | -.20** | -0.11 | -0.08 | -0.13* | -.16** | -0.05 | -0.05 | -0.07 | -0.09 | -0.04 | -0.11 | -0.03 | -0.05 |

**Table S4:** Correlations of 17 mineral elements and 15 morphological traits of NILs population of Towada brown rice subjected to cold stress.

**Table S5:** Some information about the SSR (interval) markers near the identified QTLs.

| **Minrl.** | **Chr.** | **Int. SSR** | **Motif** | **Rep** | **No.** | **Forward Primer** | **Reverse Primer** | **P Size** | **SSR start** | **SSR end** |
| --- | --- | --- | --- | --- | --- | --- | --- | --- | --- | --- |
| **Ca** | 1 | **RM5362** | **AG** | **13** | **2169** | **GAGCGCTAGGGCTTTGGATCG** | **GCCCATGTACGATGACGTGTACC** | **159** | **41080477** | **41080502** |
|  |  | RM8099 | AG | 15 | 2170 | CAAACATGGGCCAGAATTAAGAGG | GGCAGAGTGAAGAAGAGAGGAGAGG | 149 | 41088199 | 41088228 |
|  |  | RM12171 | ACG | 8 | 2171 | GTGATGCTTTCGTCGTCGTTGG | GGAGGACAAGAACAAGAACAAGAACG | 156 | 41106405 | 41106428 |
|  |  | RM12172 | AGG | 7 | 2172 | CTTCTTTGGAGCCACCGCAACC | CCATGCAATCCTCTCTTGTGTCTCC | 116 | 41107173 | 41107193 |
|  |  | RM5536 | AC | 14 | 2173 | CACGTACCAGCCTTGATGAATCC | TGGGCTATACTAATCCCGTCATCC | 175 | 41160287 | 41160314 |
|  |  | RM5310 | AG | 15 | 2174 | GGGACCAAGACCTTTCCAATGC | GCGGAAGCAGGAGAATCGTAGC | 230 | 41190755 | 41190784 |
|  |  | RM5310 | AC | 15 | 2175 | GGGACCAAGACCTTTCCAATGC | GCGGAAGCAGGAGAATCGTAGC | 230 | 41190784 | 41190813 |
|  |  | RM12176 | AT | 16 | 2176 | AAGACTAGTGGTCAAACAGTGC | AGGATGGAGGAAGTATGTATGC | 296 | 41285247 | 41285278 |
|  |  | RM12177 | AGC | 7 | 2177 | CGGAATGGGATGGAACAGAAGG | AGGGAACAAAGAGGGCGAAAGG | 256 | 41306815 | 41306835 |
|  |  | **RM12178** | **AT** | **20** | **2178** | **CCTGTTAAGGTACCGAGTATTTCC** | **GAAGCCAAACGTAGTTGTACCC** | **205** | **41309238** | **41309277** |
| **Fe** | 2 | RM12406 | AT | 34 | 113 | CATCGATATTGTGCGCATTGG | GTAGATTGTGGGTACATGTGAACTCG | 328 | 1705077 | 1705144 |
|  |  | RM12409 | ACAT | 24 | 116 | CGAATCTTGGAACACATCAACG | TGTGCCATTCATTTGGACACC | 651 | 1705915 | 1706010 |
|  |  | RM12431 | AG | 12 | 138 | CTCCGTCCCTTGGAGGTTAGC | GATGGGCCGAATTCCTCTCG | 306 | 2059556 | 2059579 |
|  |  | RM12438 | AG | 11 | 145 | ACCCGCATGCATTGTAGCTAGG | AGGTCCTCCTCCTCGGTTGC | 433 | 2160253 | 2160274 |
|  |  | RM12440 | AG | 10 | 147 | CGATCAAACTAGGTCGATGATGG | GTCGTCTACTTCCCGCAAGG | 94 | 2214318 | 2214337 |
|  |  | RM5529 | AC | 13 | 151 | GTACTACATCGGTTGTGTAGTTGG | CATACGTTAATGGCTCATCTCG | 396 | 2219467 | 2219492 |
|  |  | RM12448 | AT | 28 | 155 | CAATGGGCGGGTTTGACAGG | ACCACTTTGCTGTTCACGTCTCG | 246 | 2362397 | 2362452 |
|  |  | RM12453 | CCG | 7 | 160 | ACCTCGCCTCGGTTCAGTGC | ACTCGCTCCTCCTCCTCACTCC | 112 | 2450987 | 2451007 |
|  |  | RM12455 | CCG | 7 | 162 | GTCGTGGTGGATACCAGGATGC | GACTCCGACAAGTGGGAGGTAGG | 188 | 2576446 | 2576466 |
|  |  | RM12457 | CCG | 7 | 164 | GTCGTGGTGGATACCAGGATGC | GACTCCGACAAGTGGGAGGTAGG | 188 | 2613464 | 2613484 |
|  |  | RM12477 | AT | 31 | 184 | TCTTACTCCCTACAGGCTGATACTCC | TTGGACAGAGCACGGTCTCC | 424 | 2798194 | 2798255 |
| **Fe &** | 6 | RM19487 | AG | 12 | 262 | TTGTGTTTGGCTCGGGAATTGG | TGTGTTTCTTCGCTTGCCTTGG | 114 | 4245918 | 4245941 |
| **Cr** |  | RM19489 | AT | 42 | 264 | TAGGTGGACACCCATCCATTTATTGC | CCTGCCGCTCCTCTCTTCTATCG | 448 | 4283379 | 4283462 |
|  |  | RM19491 | ATGC | 5 | 266 | CCAAGTGCTTGTTTGGATCTTACC | CATCTCACTCTTCTTCCTCGAACG | 192 | 4285498 | 4285517 |
| **Fe,** | 6 | RM4608 | AT | 23 | 269 | ACCCAATATGGTGCAATAGAGACC | CACCTCCACCAACTATTGACAGG | 397 | 4315069 | 4315114 |
| **Zn &** |  | RM2434 | AT | 45 | 270 | ATTGAGGTGGACAAGAAGGGATGAGG | CGAGTTGGGAGGAGGTGATGAAAGG | 499 | 4322794 | 4322883 |
| **Mg** |  | RM19496 | AGC | 7 | 271 | CAGATAAGGAATCAACGGAAGGTAGC | CCTCGCTCTTCTACTGGTTCTTCG | 244 | 4363776 | 4363796 |
|  |  | RM19498 | ACCC | 5 | 273 | CACGCAGACTACTCGACCACACC | CTCGTACTGGAACGGCCACTCG | 380 | 4373567 | 4373586 |
|  |  | RM6773 | AGG | 8 | 274 | GCTGCTCCACCTTCACCTTCC | CGATGGTGTGTTGTTTGGTTGC | 130 | 4416851 | 4416874 |
|  |  | RM6119 | CCG | 8 | 276 | CGTCTTCTCGAACTCTCCATCTCG | CCGATAGATCTAATCCCGCAAGG | 256 | 4439561 | 4439584 |
